# Analectic

**Published:** 1835

**Authors:** 


### MISCELLANEOUS INTELLIGENCE.

### ANALECTIC, ANALYTICAL, AND ORIGINAL.

### I.—ANALECTIC.

*Ad Sylvas Nuncius.*

1. *On the Employment of Sheet-Lead as a Dressing to Promote the Cicatrization of Wounds and Ulcers.* By Reveille-Parise.—The method of dressing with sheet-lead, appears to me much better adapted to effect the object proposed, than the ordinary means. The plan adopted is, to cover the wound, already in a condition to heal, with a piece of sheet-lead, of an extent and thickness to be determined by the size of the diseased surface, and other circumstances to be mentioned presently. It should be properly confined by means of a compress and bandage, or by strips of adhesive plaster, when the bandage alone is not sufficient. There is no species of dressings more simple, expeditious, comfortable and convenient than this. The softness and pliability of the lead, allow it to adapt itself readily to the shape of the parts, while the same properties render it quite as easy to cut it of the proper shape and dimensions, as an ordinary piece of lint, and at the same time make its application exceedingly easy. That the metal used in this way is innocuous, we think will not admit a doubt, since the deutoxide of lead, or litharge, has been long employed for the purpose of cicatrizing wounds and ulcers. It must not be inferred, however, that the sheet lead, like these substances, acts in virtue of any specific properties; its operation is purely mechanical. That this is the case, is evident from the fact, that I have obtained the same effects from the employment of plates of tin, silver and gold. The ductility of the lead, however, and its cheapness give it the preference.

An enumeration of all the advantages of this method of dressing, will suffice to show that it cannot be necessary to resort to unguents.

What, in effect, can such applications accomplish? The object is not to calm irritation and promote suppuration, but to obtain cicatrization. As the adherence of the borders of the wound is not to be dreaded, applications of unguents and cerates are altogether useless. The non-adherence of the dressings to the edges, and to the diseased surface, is an important advantage; because there is no pain experienced in the removal of the dressings; the newly formed cicatrix is not liable to be torn up, and the wound is kept clean and free from crusts and particles of ointment and charpie, which are apt to adhere to it when other dressings are employed.

I shall not swell these observations by a detail of individual cases; a few examples, however, may be cited in order to illustrate the general precepts. M. de S. in descending a stairs, received a slight wound in the heel, which refused to cicatrize. The application of ointments and plasters increased the irritation and swelling. Fine charpie, at first dry, afterwards endued with cerate, adhered to the surface, and even produced greater aggravation than the other dressings. At this juncture I was called. A piece of sheet lead was confined upon the part by adhesive strips, and the ulcer cicatrized in a few days.

These dressings can be applied with exceeding ease and promptitude, and they need not be so often repeated as those in common use; since they are not attended with that heat and humidity of the wound so favorable to decomposition, nor with itching irritation so apt to be excited by unctuous substances. It is easy, therefore, to conceive how by this procedure the healing process will be promoted, there being nothing to interrupt its progress. The operations of nature, when not thwarted in their tendency, and when aided by the co-operation of art, set aside the obstacles to the healing process, and advance steadily in the accomplishment of the cicatrization of ulcerated surfaces. These powers are never more clearly displayed than when the lead dressings are employed.

To these advantages may be added another: in the employment of the plates of lead, those formidable infections are never to be dreaded, which sometimes take place in using old and inferior charpie. Economy is likewise a consideration of some weight, the more especially, as the price of the best charpie is daily increasing, and even at present, it is a source of such heavy expense to large establishments, that attempts have been made to diminish this heavy expenditure. The means which I indicate, is as simple as it is effectual. A single plate of lead, if not too thin, will not only serve for the entire cicatrization of one ulcer, but may be afterwards employed on others,—the only precaution necessary being to cleanse it carefully, and slightly polish its surface.

These are the advantages of the mode of dressing ulcers which I propose to substitue for that in common use. They have been exemplified by the numerous applications I have made of the procedure in question, as well as by the experience of others, who since the publication of my first memoir have adopted it. So that theory and experience, in this particular, are in full accordance with each other.

At the time of my first publication, some surgeons, supposing that the leaden plates were only proposed for the cure of ulcers, did not resort to them for other purposes, and consequently obtained but partial or no success. Such was not my intention. My object was to show, that wounds and ulcers in the way to cicatrize; viz: those which are wide, superficial, red, in a state of free granulation, covered with a fibrinopurulent secretion, and surrounded with a deep rose-colored areola, should be dressed and cicatrized by the application of plates of lead. It is only necessary to remove the dressings every two or three days, according to the abundance of the secretions.

On the other hand, when the wound is deep and presents no disposition to heal—when there is pain, profuse suppuration, especially if kept up by any peculiar virus, and when it is judged necessary to resort to local applications, as cataplasms, I question whether the method in question will prove successful: such at least is the result of my own experience. Nevertheless, it should be employed as soon as these obstacles are removed, and the part manifests a tendency to cicatrize. Notwithstanding the species of extension presented by many wounds, there is reason to believe that this plan of dressing may be employed in a majority of wounds. I may even subjoin, that there are cases, in which it will succeed in a manner somewhat different from that expressed above. Thus in many abscesses and encysted tumors, after the contents have been evacuated by a small puncture and the application of a cup, if a tolerably thick plate of lead be applied to the surface, and confined by a proper bandage, the adhesion between the walls of the cavity may be obtained in a short time.

In all cases in which the diseased surface has been reduced to the condition of a simple wound, and the obstacles to cicatrization have been removed, the method proposed is superior to all others, and success is certain. It is nevertheless more particularly applicable to such as are superficial and present a great extent of surface, as is exemplified in burns. If they are slight the plates may be resorted to immediately; but when they are profound, time should be allowed for the accompanying accidents, and the profuse suppuration to pass away. These rules attended to, they all heal promptly under the application of a simple plate of sheet-lead, to be changed according to circumstances. The same may be remarked of certain blistered surfaces, in individvals of an irritable habit, and laboring under protracted diseases. Many practitioners have remarked, that such persons support with difficulty the application of charpie, adhesive strips, and plasters spread with cerate, to the raw surface. Each dressing is attended with acute pain, the surface bleeds more or less freely, and in some cases, fever and wakefulness are the result of these painful dressings, which always retard the process of cicatrization. All these inconveniencies may be effectually obviated by the simple application of a piece of sheet-lead.

This method of dressing is equally successful upon parts in which cicatrization is with difficulty accomplished; as the elbow, the crest of the tibia, the malleolus and the tendo achilles. Few surgeons have escaped the embarrassment experienced in healing ulcers of small extent upon these parts; by the new method they may be cicatrized promptly.

Dartrous ulcers, and indeed all dartrous and erysipelatous affections in a state of suppuration, cannot be healed by this method; but are ameliorated by it, when it is not found necessary to employ tonic applications, or when continuous medicated lotions are simultaneously used. Experience has demonstrated, that there are wounds with loss of muscular substance, which cannot be cicatrized by any means. The plates of lead, under such circumstances, have not appeared to me to be more successful; yet they can be applied with more convenience and promptitude than dressings of charpie. These advantages are greater than might be supposed in such wounds, which are thus covered with a kind of protection, capable of serving as a substitute for the natural integuments.

It is universally known, that extensive cicatrices become lacerated with extreme facility, and that they afterwards heal with difficulty, either on account of the feeble vitality of the part, or some other cause. Hence the disadvantages of dressings of charpie and cerate; each successive removal of them tending to destroy the newly formed cicatrix, and in this manner, to perpetuate the sore. These inconveniencies are removed by the lead, though the cure cannot be so soon accomplished as in other cases. Even when the new cicatrix has formed, it may be strengthened by the lead; and it rarely becomes torn a second time under this species of protection.

The advantages of this new mode of dressing are fully signalized in wounds, ulcers, and erosions which take place in limbs affected with œdema or other species of engorgement. Experience has fully shown the insufficiency and even danger of charpie and cerate dressings in such cases, and their inevitable tendency to aggravate the disease. It may be replied, perhaps, that if seconded by compression, they will prove successful; but to this it may be objected: 1st. That compression does not diminish the inconveniencies of the application of charpie and unguents to the wound. 2d. That this compression is not always practicable. 3d. That the lead dressings, instead of opposing any obstacle to the employment of this means, favors, on the contrary, the attainment of its good effects. My experience has abundantly proved the truth of this assertion. The following case affords conclusive evidence. The Baron L——, of an active temperament, but broken down by age and the labor of the cabinet, was attacked with a chronic enlargement of the liver, which ended in dropsy, and finally destroyed his life. During the course of this protracted disease, his legs became tumid, even before symptoms of serous infiltration were apparent; the skin became red, sensitive, preternaturally thin, and was affected with frequent excoriations, which terminated in ulcers difficult to heal. These effects were produced by the friction occasioned by fine thin stockings, and his legs were covered with ulcers, which added to the pain of his situation. The finest charpie, the freshest cerate, and the most soothing dressings increased the mischief. Compression was also employed without success, and was so painful that it could not be supported. I placed the legs in a horizontal position, covered the ulcers with a thin plate of lead, and confined it with a moderately tight bandage: the diseased surface was soon healed, and as other ulcers formed they were treated in the same manner.

In some young children, blistered surfaces are very prone to ulcerate profoundly, and to increase in extent, in spite of all local applications. Fresh butter, cerate, goulard lotions, etc. often prove of no avail; but a simple plate of lead will often heal them in a short time. I was called to visit a child, to whose arm a blister had been applied for the relief of an attack of bronchitis. The blistered surface was so extensive, notwithstanding all the means that had been employed, that nearly the whole arm was excoriated. The child was also affected with acute pain, want of sleep, and extreme irritation. I covered the whole surface with a thin leaf of lead, which was removed next day, and a cure was speedily effected. It also often happens, that issues, and cauteries, after being established, cannot be sustained, on account of the preternatural irritation of the dermoid tissue, or some other inappreciable cause. The contour of the part is surrounded by an erysypelatous blush, which is augmented under the use of ivy leaves, cautery paper, and other means usually employed in such cases. The application of a thin plate of lead for a time more or less considerable, remedies all these difficulties. In confirmation of this assertion, I could cite numerous examples, did my limits permit.

As a dressing to ulcers, properly so called, the plates of lead can scarcely be employed, except on the score of economy, and not for the purpose of promoting cicatrization, as long as the ulcer is perpetuated by some special cause, either local or general, which must be first removed. Varicose ulcers are, perhaps, those in which this remedy promises most advantages, especially when aided by pressure. Sheet-lead was employed successfully in these cases, by M. Marjolin, (Dict. de Med. Art. *Ulcere.*) By some practitioners, the method of Baynton, or the application of adhesive strips, is deemed preferable to the sheet-lead. I would reply: 1st. That this last method and the leaden plates are not employed under the same circumstances, and for the reasons enumerated above. 2d. That there are cases in which Banyton's method is impracticable; as, for example, when a leg is much swollen and infiltrated, and at the same time ulcerated at several points,—also, when the ulcer is seated upon any other part than the extremities.

The following are the rules I observe in the application of this method of dressing. I will suppose that one of the extremities has received a severe wound. After having examined its depth and extent, with the object of ascertaining that there is neither fracture nor dislocation, and removed all extraneous matter, should any exist, I attend to the disgorgement of the wound, either by suffering it to bleed freely for some time, by means of incisions, and especially by the repeated application of a cup with a piston attached. This latter procedure greatly favors union by the first intention, where it is practicable, and facilitates the healing of other wounds. The parts having been thoroughly disgorged, the subsequent inflammatory congestion should be combatted by the application of compresses dipped in cold water, or goulards lotion, either with or without the addition of laudanum, according to the intensity of the pain, and the susceptibility of the subject. When inflammation is fully developed, it only remains to moderate the organic excitation, and to counteract the local determination. All practitioners resort very properly, under such circumstances, to emollients in form of cataplasms, fomentations, embrocations, etc. which are continued until suppuration is established. This process was formerly divided into two periods: 1. *Preparative* suppuration, when the pus is serous and crude, to use the language of the times. 2. *Consolidating* suppuration. It would be better, perhaps, if this distinction were not neglected at the present day, since it is just, and is founded in fact. During the continuance of the first, I employ cataplasms, digestive pledgets, cerate, and other analogous means; but so soon as the second period arrives, I abandon the use of charpie, and all unctuous applications, thinking with Ledran, that it is not by medicaments introduced into a wound that it is cured, and that strictly speaking, all applications of charpie, or medicaments, should be regarded as a foreign body. It is under these circumstances that I resort to the sheet-lead, according to the regulations above described, which I continue until the wound is completely cicatrized, and some time afterwards, with the view of furnishing a salutary degree of support. This dressing should only be removed every second, third, or fourth day, according to the abundance of the suppuration. When it is necessary to use pressure, the piece of lead may be a little thicker than ordinary, taking care not to have it so heavy as to occasion fatigue by its weight. Should the granulations become exuberant or spongy, and the surface of the sore remain stationary, the ordinary means must be employed.

I could cite the testimony of many surgeons in favor of this method of dressing ulcerated surfaces, but shall content myself with that of M. Yvan, surgeon in chief of the *Hotel des Invalides.* He not only employed the sheet-lead dressings with success in the cases indicated by me, but likewise in the treatment of hospital gangrene.—*Bulletin generale de Therapeutique.—Arch. of Med. and Surg. Sci., June,* 1835.

2. *Experimental Trials on the Efficacy of repeated Purgatives in Typhous Fever.*—Hamilton, of Edinburgh, was, we believe, the first physician who insisted on the efficacy of purgative medicines as a principal means of cure in typhus fever. His doctrine has been adopted by many practitioners; but by others, and particularly by the French, it has been combatted as a method which is incompatible with the pathology of that affection. In order to afford means for deciding this question, important for the value of the truth which affirmative decision may establish, and interesting from the degree of attention which the subject has so long excited. M. Piedagnel undertook a series of experiments at the *Hotel Dieu,* which were calculated to develop an answer to it, and he certainly has conducted them in a manner which is well designed for determining the true value of the purgative method. All the typhoid fever cases which he treated, were, without any distinction of symptoms, period of disease, etc., submitted to the action of purgatives during *the same season.* The number of patients experimented on was large, and no other active remedies, such as bleeding and leeches, were applied, these being avoided in order that the result might be as positive as possible. We now present our readers with an analysis of the paper on this subject, which M. Piedagnel has published in the 13th No. of the French *Gazette Medicale,* the paper having been read at the Academy of Medicine on the 24th of March.

The author distinguishes four kinds of typhus fever, viz:

1st. Simple typhus.

2d. Adynamic typhoid fever; to the common symptoms are superadded those arising from a considerable alteration of the intestinal canal, consisting in numerous ulcerations of the mucous membranes: here the abdominal affection becomes prominent, and the patient at the end falls into a state of adynamia.

3d. Ataxic typhus: here the cerebral symptoms predominate; there is a peculiar delerium, pain in the head; the senses are more or less perverted, and the muscles contract, etc.

4th. Putrid typhus (fievre typhoide foudroyante;) this form can only be likened to the effects of poisoning; in three or four days the patient dies, and the autopsy does not reveal any organic alteration.

Such are the very different forms of typhus fever which M. Piedagnel has submitted to the following treatment.

On the day after the patient's reception into the *Hotel Dieu,* the purgatives were immediately administered, when the symptoms were severe; if not, he was allowed to remain quiet for one or two days. When the treatment was commenced, a purgative was given every day, or every second day, according to circumstances. The patient took for drink, water sweetened with syrup of currants, and his diet consisted of three *bouillons* (weak broth) per day. This regimen was invariably pursued; and the modifications of the treatment were very simple. Thus when a patient went naturally to stool, a slight purgative only was administered. In cases where a gentle purgative produced no effect, a stronger one was immediately given. The rumbling sound of the bowels, and particularly the appearance of meteorismus, were an indication for the employment of purgatives still more energetic. These means usually produced six to ten stools in the twenty-four hours. In some cases the patients were purged only once or twice during the whole course of the disease; in others ten or twelve times; but in general three or four purgative doses were sufficient. The state of the abdomen never furnished any contra-indication; thus a severe pain in some one point of the abdomen generally yielded to the first or second purgative, and never resisted the third. The purgative medicines employed were Eau de Seidlitz, from two glasses to one or two bottles; a solution of one or two ounces of Epsom salts; castor oil; calomel; and croton oil.

On comparing the results obtained by M. Piedagn'el with those obtained in the different hospitals of Paris, they certainly present a very favorable aspect.

From the 1st of June, 1834, to the 1st of March, 1835, no less than 134 cases of typhus fever were treated by the author at the *Hotel Dieu*, all exhibiting in a greater or less degree the peculiar expression of the face, state of the mouth, rales moquex and sibilant, diarrhœa, pain of abdomen, petechiæ and sudamina, which distinguish that affection.

The cases may be arranged under the following categories:

1st. *Simple Typhus.*—69 cases. No death. Mean duration of the disease 20½ days. Mean duration of treatment 13½ days. Average number of purgatives 3½.

2d. *Adynamic Typhus.*—49 cases. Cured, 39; dead, 10. Mean duration of the disease 17½ days. Mean duration of treatment 10¼ days. Average number of purgatives 3.

3d. *Ataxic Typhus.*—16 Cured, 7; died, 9. Mean duration of the disease 29 days. Mean duration of treatment 19 days. Average number of purgatives 6⅓.

Hence in 134 cases, we find 115 cured, 19 dead, giving the proportion of mortality as 1 to 7, 1-19th of the cases treated. But amongst the 19 deaths, M. Piedagnel enumerates two which ought not strictly to be included; one, cured of the fever and on full diet for four days, was cut off by a double pneumonia; the other, also cured, contracted the small-pox, which terminated in death. If we abstract these two cases, the general mortality will be very nearly 1 to 8. For adynamic typhus the proportion is 1 to 4 9-10ths,—the author of the memoir says 1 to 3 9-10ths, but he is evidently mistaken. Finally, he enumerates amongst the ataxic cases, the only two examples of *fievre foudroyante* which presented themselves in the course of the year; hence in this severe form the cures and deaths may be accounted exactly equal.

Let us now compare these proportions with the result of the practice of MM. Chomol and Bouillaud at the *Hotel Dieu* and *La Charite*.

*Hotel Dieu.*

| | Patients. | | Dead. | | |
|---|---|---|---|---|---|
| In 1830 . . . . | 27 | — | 8 | — | 1 to 3.375 |
| 1831 . . . . | 56 | — | 16 | — | 1 to 3.5 |
| 1832 . . . . | 23 | — | 5 | — | 1 to 4.6 |
| 1833 . . . . | 30 | — | 10 | — | 1 to 3 |

*At La Charite.*

| | Patients. | | Dead. | | |
|---|---|---|---|---|---|
| In 1834 . . . . | 31 | — | 5 | — | 1 to 6 1-6 |

Thus in the practice of M. Chomel the mortality is as 1 to 3.4871794. In that of M. Bouillaud, or rather in the small number of cases reported by him, 1 to 6.2: and in that of M. Piedagnel as 1 to 7.0526315789 47368421. (The decimal runs to this great length before it begins to repeat.)

Hence the author concludes that so far as regards the mortality, the treatment of typhus fever by purgatives is superior to any other practised at the present day; but it is extremely fatiguing for the patient, and requires extreme care on the part of the physician. The most frequent complications with which it may be reproached, are inflammations, which sometimes determine death; but, on the other hand, we very rarely find extensive gangrene, abscess, meteorismus, etc. and the convalescence is probably less prolonged.—*Lancet.*—*Bost. Med. and Surg. Jour., June,* 1835.

3. *On the Cure of Ascites by Stimulating Injection.*—In 1832, a favorite blood-hound bitch, the property of a friend, became dropsical, and had ascites. She was tapped, and a strong stimulating injection thrown into the peritoneal sac immediately after the evacuation of the fluid from tapping, considerable inflammation supervened, but the antiphlogistic measures adopted were successful in overcoming it, and a perfect recovery took place, the bitch never having any return of the disease, and frequently afterwards had young. After this, I was led to reflect on the benefit that might be derived from a similar mode of treatment, where the disease occurred in the human subject, and more readily came to the determination of trying it on recollecting the example although on a smaller scale, of curing hydrocele by a stimulating injection. The great objection offered to attempting the cure of acites after this mode, has been the fear of inducing an extensive and fatal inflammation of the peritoneum, but it did not appear to me to be attended with the quantum of risk so generally apprehended as serous membranes, however liable to be excited into an inflammatory action in a healthy state, are by no means so easily as when in that peculiar state wherein the serum is so superabundant as to form dropsy. On the contrary, they require the application of such remedies as will decidedly create an increased action, and will bear stimulating applications without injury to a much greater extent than we have hitherto allowed ourselves to believe. Good, in his "Study of Medicine," says Paullini relates a singular mode of operation, "the patient not submitting to the use of the trocar, had the good fortune to be gored in the belly by a bull, the opening proved effectual and he recovered."

The only conclusion we can come to on perusing such a case, is that the bull's horn having entered the peritoneal sac, allowed the collected fluid to flow out, and merely created such an increased action throughout the weakened peritoneum as restored it to healthy and vigorous action, whereas had the peritoneum been similarly wounded when in health, the probability is that the action would have been so much increased as to have produced violent inflammation, rapidly terminating in death. In consequence of the train of reasoning I had adopted, I determined on trying a stimulating injection for the cure of ascites in the first favorable case, and the result of which I have now to detail:

In 1823, a negro slave attached to Williamsfield estate, by trade a cooper, elderly but healthy, was suddenly seized with general dropsy, a variety of remedies were used, and the general dropsy disappeared, but fluid was evidently collecting in the belly, the disease rapidly increased in spite of all the usual remedies, and soon required that he should be tapped for the relief of the more urgent symptoms. I tapped in presence of my friend Dr. James Rankine, now in extensive practice in England, and after drawing off a very considerable quantity of fluid, a quart of cold water in which I had mixed a wine-glass full of house rum, was thrown into the peritoneal sac; the patient was then placed in a recumbent posture, and means taken to have the injection applied as generally as possible to the whole of the sac, the patient stating in a short time that he felt some degree of pain and uneasiness, as much of the injected fluid as possible was drawn off, but certainly not so much as was thrown in. I watched the patient most anxiously, and the morning after the operation he complained of considerable uneasiness and some pain on pressing the abdomen, there was no fever or any material affection of the pulse. I had recourse to fomentations, and before evening all uneasiness had vanished. Suppuration to a small extent took place in the wound made by the trocar—the patient was put under a course of tonics, and good diet with gentle exercise; his recovery was rapid, and he resumed his usual work on the estate, and continued to labor for six or seven years, and then died of a disease not connected with his former dropsical attack. I regret I had not an opportunity of examining the body after death. The remarks I would now make on this case are few, it being a solitary one prevents my drawing a conclusion that further trial may prove to be fallacious; yet I cannot help feeling sanguine as to the ultimate success of curing ascites by means of stimulating injections when tried under favorable circumstances as readily as hydrocele is now cured by similar means. It can only be tried with advantage or hope of success in those cases where there is no extensive derangement of any of the chylopoetic viscera; where it arises in dirt eaters, with whom there is generally disease of the stomach, liver and spleen, or in drunkards from indurated liver and lost powers of digestion; it would probably prove of no avail; where ascites supervenes, or debility from loss of blood or long continued fever, and from other causes not connected with visceral disease we may anticipate success from stimulating injections thrown into the peritoneal sac after tapping. At all events the above case will show, that a stimulating injection may be used without inducing the dangerous symptoms, the dread of which has hitherto prevented its being tried. I am not aware that any British surgeon has before this case, tried the effects of a stimulating injection in similar cases, and I shall feel proud indeed, should the case now recorded, be the means of future benefit to dropsical patients.—*Jamaica Physical Journal.*—*U. S. Med. and Surg. Jour.*

20th March, 1835.

4. *Hypertrophy of the Muscular Coat of the Stomach.* By Dr. OTTO. —The records of science, as yet, contain but few examples of this pathological phenomena. Neither Morgagni, Haller, nor Baillie allude to it. Meckel, in his Manual, states, in a general way, that in great eaters the muscular coat of the stomach is often thicker than is usual. M. Louis has indeed detailed two well-marked instances of this hyperthropy which he met with; in both, the pyloric orifice was affected with scirrhus and he was thereby led to regard the former change as the result of the latter. A woman, 49 years of age, of a spare habit of body, had long suffered from mental anxiety, which she vainly endeavored to subdue by immoderate indulgence in the pleasures of the table; —in course of time the appetite became quite voracious. Her chief malady was a difficulty of breathing, which returned periodically every evening, and was accompanied with the sensation as if a heavy ball was rising from the lower part of the belly to the region of the heart, and there stopped, and obstructed the respiration. These symptoms lasted generally for about six or eight minutes, and then gradually subsided. Each attack became more severe; and at length they sometimes induced delirium. A general emaciation supervened, although the bulimia was not diminished. All the remedies tried to relieve her failed, and she died in the course of a few months from marasmus.

*Autopsy.*—Thoracic organs healthy; omentum almost entirely wanting; liver, gall-bladder and spleen natural. The stomach was puckered, and evidently much thickened, especially towards the pyloric orifice, and along the great curvature; its blood-vessels were empty: the mucous membrane, coated with a viscid deposit, was thin, transparent, but not softened; its surface was elevated into bold irregular projections, by the subjacent bundles of muscular fibres, extending from the great cul-de-sac towards the pylorus; between these projections were hollows, or depressions; and thus this stomach presented an appearance not unlike to that of the inner surface of the heart. The thickness of some of the muscular fasciculi was at least half an inch. The rest of the alimentary tube was normal.—*Archives Generale, from Hufeland's Journal, Feb.* 1833.—*Amer. Jour. Med. Sci., May,* 1835.

5. *Existence of Charcoal in the Lungs.*—Dr. G. PEARSON, in a memoir in the Philosophical Transactions, for 1813, on the coloring matter of the black bronchial glands, establishes the fact, that a dark carbonaceous matter accumulates to a considerable extent in the above mentioned glands, during the course of life, in the human subject, which he thinks is introduced into the lungs with the air in breathing. The black matter was obtained not from the red glands, but from such only as were dark-colored, by acting on them indifferently with caustic potash or nitric acid, so as to dissolve or destroy the organic matters. This black matter proved to be a tasteless and infusible powder, insoluble in muriatic acid, nitric acid, and perhaps all common acids except sulphuric; affording as large a proportion of carbonic acid, when burnt, as animal or vegetable charcoal which has been dried at the same temperature, and equally unaffected by heat in close vessels. In fact, although not completely freed from animal matter by Dr. Pearson, the black powder possessed all the properties of carbon or charcoal, as we have it in lamp-black or soot.

Dr. Pearson believes that this sooty matter is deposited on the surface of the bronchial tubes and air-vessels from the air inspired; and, from a comparison of the black lines and black net-like figures, many of them pentagonal, on the surface of the lungs, with the plates of the lymphatic vessels, by Cruikshank and others, which they exactly resemble, he infers "that the lymphatics of the lungs absorb a variety of very different substances, especially this coaly matter which they convey to the bronchial glands, and thus render them of a black or dark blue color. When a thin slice of the surface of the lungs was treated with nitric acid, these black lines remained, as black threads of charcoal, after the solution of the other parts.

Dr. Graham, of Glasgow, has had several opportunities of substantiating the existence of a carbonaceous matter, in a state of extraordinary accumulation, in black lungs, and the *Edinburgh Medical and Surgical Journal*, for October last, contains an account of his investigations. From these he draws the following conclusions:—

1. The black matter found in the lungs is not a secretion, but comes from without. The *pigmentum nigrum* of the ox I find to lose its color entirely, and to leave only a quantity of white flocks, when rubbed in a mortar with chlorine water. *Sepia*, which is a preparation of the dark colored liquor of the cuttlefish, was also bleached by chlorine. But the black matter of the lungs was not destroyed or bleached in the slightest degree by chlorine. It even survived, unimpaired, the destruction of the animal matter of the lung by putrefaction in air.

2. This foreign matter probably varies in composition in different lungs, but in the cases actually examined, seems to be little else than lamp-black or soot.

3. It is evident that the accumulation of charcoal in the lungs, which Dr. Pearson remarks to occur more or less in every subject, may proceed in peculiar circumstances to a great extent, without affecting immediately the general health of the patient. In Dr. Laurie's and Dr. Buchanan's cases, all the patients were robust and healthy, had no cough, and no expectoration of black matter.—*Amer. Jour Med. Sci. May*, 1835.

6. *Case illustrating the Anomalous Nervous Symptoms, occasionally induced by Tœnia.* By JOHN SCOTT, M. D.—In the spring of 1830, I was requested to visit James Simpson, a pastry-cook, residing in Rose street. He had been laboring under symptoms of disordered stomach for a year and a half, and for the last nine months of that period the disease had assumed a serious form, he having been constantly affected with vomiting about an hour and a half after taking food, whether it was solid or fluid, unless in the very smallest quantity. He was also, from his wife's account, liable to fits, the nature of which I could not ascertain from her description. He had been under a most respectable practitioner during the whole period, and used a variety of means without benefit.

The color was sallow and unhealthy; the skin clammy; pulse soft and natural; some emaciation, but not to a great extent, or in proportion to the excessive weakness of which he complained; bowels very irregular; usually constipated: constant feelings of pain and gnawing in the epigastrium; not much increased on pressure. Different medicines, chiefly of the laxative kind, were used, when a smart attack of bowel complaint came on, during which a considerable quantity of blood, mixed with feculent matter, was passed, and then the vomiting ceased; the uneasy feelings disappeared, and he considered himself as well.

In about two months afterwards I was requested to see him again, when I found that the vomiting after food had returned, and that the bowels had become very constipated. He complained of pain over the whole head, which was particularly intense on the fore-part, increased by light and motion; the pupils were contracted; the pulse ninety-two, full and hard. There were intervals of ease, and he had occasionally a quiet but short sleep. Laxative medicines were given, and blood taken freely from the arm, with instantaneous relief, although of short duration, for the next morning the pain was as severe as ever.

A friend saw him with me, and we were somewhat puzzled whether to consider it as inflammation of the brain or not; but considered it prudent to repeat the bleeding. The appetite was gone, great thirst, but the liquid taken was immediately vomited. His wife said the fits had returned. It is unnecessary to enter into long details. In a few days the pain in the head was somewhat mitigated, though he still complained much of it. It was in visiting him early in the morning that I first witnessed what were called his fits. He lay in his bed with his eyes open and directed upwards. When I inquired how he felt himself, he did not look at me, but replied, "Quite well,—there is nothing the matter with me." "How is your head?" "Quite well." "No pain?" "No." "Any sickness?" "No." The pulse was calm and about ten beats lower than usual. When conversing with him he suddenly rose up in the bed, gave a long yawn, stretched out his arms, gazed at me for a moment, and then put his hand to his head, and complained of the pain in it.

A few days afterwards I found him dressed by the fireside, and scolding his wife for some alleged fault. When I inquired what the matter was, he said he had been very ill used, and that he had dressed himself to go and complain to Dr. Scott, and entered into long details, addressed to me as a third person, perfectly unconscious of my presence. The eyes were not directed to me but straight forward; and he always said he was quite well. On starting from this state, which was almost instantaneous, he complained of his head; the vomiting still continued; and he had a constant gnawing feeling in the epigastrium. I tried a variety of means, and witnessed several of the attacks, and there was one remarkable circumstance connected with them, that when awake he could scarcely be persuaded to take food of any kind, while in his unconscious state he complained bitterly that his wife had been starving him, and would give him nothing to eat, and when food was offered he took it eagerly. His natural sleep was rather sound, and he awoke in the usual manner; but generally in the morning relapsed into fits of unconsciousness, which continued from ten minutes to a quarter of an hour, and sometimes half an hour, and recurred three or four times a day. When questioned as to his sensations, he professed his utter unconsciousness of having been spoken to, or of my presence. Previous to his waking he frequently rose from his bed, dressed himself, walked about, and was with great difficulty prevented from going into the streets, generally for the purpose of visiting me. It now occurred to me that these strange appearances might be connected with worms, I therefore gave him a full dose of turpentine, followed by castor oil, which brought away many pieces of *tœnia solium*, with much mucus. The dose was repeated several times, the worm discharged, and all the symptoms disappeared. He has had frequent returns of sickness, and other indications of his former disease, but these have been invariably removed by turpentine or decoction of the pomegranate root, and followed by a discharge of pieces of worm. For the two last years he has been perfectly well.—*Edinburgh Med. and Surg. Jour.*—*Ibid.*

7. *On the Organization and Origin of Intestinal Worms.* By Wm. Stokes, M. D.—(Extracted from his lectures on the theory and practice of medicine, delivered at the Medical School, Park street, Dublin.)—There are few subjects which possess so much interest in a physiological and pathological point of view, as that of intestinal worms; and in order to have correct notions, it will be necessary to be acquainted with the investigations of modern science on this subject. Worms are found in most classes of animals. They occur in reptiles, fishes, birds, in the different classes of quadrupeds, and in man. In man they do not exist in such abundance, nor so frequently as they do in birds and fishes. With respect to their places of habitation, we find them, first in cavities which have an external communication, and next, in the parenchymatous substance of organs; and we generally observe, that those which inhabit the cavities are different from those met with in parenchymatous parts. We observe, also, that the species existing in the different organs and cavities are not only different in their nature, but that there is a difference between the worms which inhabit separate portions of the same organ or cavity. In one part of a cavity or organ we find one species, in another a different, and this occurs almost invariably, as if it was regulated by a fixed law of the economy. A peculiar species of worm, occurring in man, called the *Distoma hepaticum*, is never found except in the liver or gall-bladder. If this animal had been introduced from without, it would certainly be detected in some parts of the intestinal canal, but this is never the case. Rudolphi states, that the *Strongylus horridus* is to be met only in the œsophagus of aquatic birds, and the *Ascaris obtusa* in the stomach of mice.

Generally speaking, worms are of three different forms—cylindrical, riband-shaped, and vesicular. Their organization varies from the lowest scale, in which we can scarcely trace, as it were, the rudiments of an animal; beginning with the tape-worm, which presents little more than a cellulo-gelatinous mass, we ascend gradually until we arrive at a high degree of organization, where we find well developed muscles, a difference of sex, generative organs, and, according to some anatomists, a tolerably perfect nervous system.

Now, to remove all sources of doubt and error on this interesting subject, and to establish proper principles of treatment, let us examine into the origin of these animals. I shall confine myself to the consideration of the origin of those worms which inhabit the human intestines, as they are the only species which we have to do with as practical physicians.

You will at once perceive that worms must be derived from one of two sources; either as introduced from without, or formed originally within the bodies of man and other animals. It is maintained by those who are in favor of the first supposition, namely, that they are introduced from without that similar animals are to be found in the external world, and that they are introduced either in the form of ova, or in a state of perfect development, with the food or drink, or by the respiration of the animal. Observe, this doctrine is founded on the validity of the assertion as to whether animals similar to intestinal worms are to be met with in external nature. Linnæus states that he found the tape worm, and the small ascarides, a species now called *oxyuris vermicularis*, in a marsh in Lapland: but Muller, a much more accurate helminthologist, has since shown, most satisfactorily, that Linnæus was completely mistaken, and that those he had observed are never found to exist within any animal whatever. There are many observations on record similar to those of Linnæus: but as they were made at a time when natural history was in its infancy, and as they have been disproved by the researches of modern zoologists, I shall not notice them. I believe there is no well-authenticated instance on record of tape worms, lumbrici, or ascarides being found living in any situation external to the animal body. Every one of you, gentlemen, have seen worms in the intestinal canal, or recently discharged by stool or vomiting, but I will venture to say, that not one has ever observed them in any article of food, in earth, or in water. Bremser, who is a high authority, makes a very pertinent remark on this subject. "We find," says he, "all animals most abundant in that situation which has been assigned to them by nature. Now, if these animals were accidentally introduced from without, we ought to find them more abundant in the earth, water, etc.; but the contrary we have seen to be the fact."

But it is contended that these animals may have been introduced from without, and that in consequence of a change in situation, nutriment, and other circumstances, their forms may be altered; and it is argued in support of this hypothesis, that external circumstances will and have been observed to change the forms of plants and animals in a very remarkable degree. In addition to this it may be said that an alteration in the nature of its food may even produce an actual change in the function of the animal. It is a singular fact that neuter bees may be made prolific by changing their food; it is shown that when a queen bee dies or is lost, the neuter bees take a grub of their own species in place of her, and by feeding it in a particular manner, it becomes capable of laying eggs.

Now supposing that intestinal worms are introduced in the form of ova into the human body, there is no reason why this sudden, remarkable, and complete change should take place. We see nothing similar to it in nature. The plant which springs from any particular seed will resemble that from which it derives its origin; the egg of any particular bird, no matter in what way it may be hatched, will produce an organized being similar to its parent. The form and character of the animal are given during the act of generation, and remain unchanged.

Again, admitting that a difference in circumstances and nutrition might produce a total change in form, it should be in our power to demonstrate the individual in the process of transition; we should find those animals in a state half between what they were and what they are, and this state we should observe of very frequent occurrence. No such thing has been ever demonstrated. Out of a vast number, Bremser did not find a single one in any stage of transition, nor has it been demonstrated by any zoologist. He also states expressly, that after having diligently examined 15,000 specimens of worms in the cabinet at Vienna, he never was for one moment at a loss to say which were intestinal worms and which were not. If there was any such transition it would have been discovered, but no such thing has ever been observed.

It appears then obvious that there is no direct evidence to prove that these animals have been introduced into the body from without, either in the form of ova or in a state of perfect development. We have nothing then, I think, but to come to the other conclusion, that they originate within the body, and this seems to be the opinion of the best physiologists and pathologists. This doctrine appears to be almost brought to a demonstration by the following facts. First, it appears that the worms which have been found in man and animals have a peculiar structure and organization, differing materially from that of the worms which inhabit the external world. This is a point admitted by almost every modern writer on natural history. In the next place we find that the worms of certain animals present peculiarities differing from those of the same species in others. Thus the bothriocephalus and tænia solium in man differ from those of other animals. You are not, however, to conclude from this that every animal has its peculiar worms, for such is not the case. Thus the lumbrici and small ascarides of man are found to exist in various animals, both carniverous and graminiverous.

It appears obvious, that if worms were introduced from without, we should not find peculiar worms in the bodies of certain animals, yet taking a certain number of different animals living on the same food and in the same situation, we find a difference in the nature of the worms which are met with in the bodies of each. Another important fact is, that worms are to be found not only in the intestinal canal, but in almost every part of the body. We find them in the cellular tissue, in the liver, gall-bladder, lungs, and trachea; in the brain, heart, kidneys, and spleen. They have been met with in the air-bladders of fishes; and Treuter states that he has found the polystoma pinguicola in the ovaries of a woman which were steatomatous, and the strongylus in an aneurism of the mesenteric artery of the horse. These animals have been observed in the anterior chamber of the eye in birds and horses, and there are innumerable examples of their occurrence in situations equally strange and anomalous. Another circumstance already mentioned, and which must be coupled with the fact just alluded to is, that there are certain species of worms which occur only in the same organs, and are never met with in any other situation.

Now observe the importance of these facts — we find that worms not only exist in the digestive tube and parts having an external communication, but also in the very substance of deep-seated viscera, and that the worms which are found in the various cavities and organs are peculiar to them. In one case we find a worm in the digestive tube, in another in the brain, in a third in the liver, in a fourth in the pulmonary apparatus, but no one has ever been able to demonstrate the trajet of a worm from one of these cavities or organs to another. It would be ideal and absurd to say in the case of worms found in the substance of viscera, that they had been introduced from without or came from the intestinal canal. The distoma hepaticus, which is found in the liver and gall-bladder, might be supposed to arrive at those situations by passing along the ductus communis choledochus, but in the various cases in which it has been found, it has never been detected in the intestinal canal, and this, I think, would not have been the case if the digestive tube had been its original situation. One of the most important facts which have been stated is, that certain forms of these animals are found invariably in certain situations, and this has been observed not only in man and other animals of the class mammalia, but also in reptiles and fishes.

In man we generally find the lumbricus inhabiting the stomach and small intestine, the tricocephalus in the cœcum, and the small oxyrus, or thread-worm, in the rectum. The preparation before me exhibits a specimen of the rarest form of worms which inhabit the intestinal canal, the tricocephalus. Here is the cœcum filled with these singular worms. The males are distinguished from the females by the whirl of the tail. If these little animals or the oxyuris had been introduced from without, we should expect to find them in various parts of the intestinal canal, but we find, on the contrary, that their situation is separate and distinct.

Lastly, *intestinal worms have been found in the fœtus both of man and other animals.* Kerking describes a fœtus, the intestinal canal of which contained a vast quantity of small worms, and another of six months, in whose stomach a large lumbricus was found. Rudolphi, Blumenbach, and others of nearly equal authority, have recorded abundance of examples of worms existing in the fœtuses of various quadrupeds, *and also in those of birds which had just broken the shell.* Those who are obstinately attached to the doctrine that worms are introduced from without, have gone so far as to assert that the ova of the worms have been transmitted at the moment of generation, a doctrine so absurd that it is unnecessary for me to enter into any refutation of it.

With respect, then, to the formation of worms in animals, we cannot help coming to the conclusion, that they are originally formed within the body, and that, in fact, there is an original generation of these animals, the result of one organization taking place within another,—the production, in fact, of a distinct being. This idea does not appear so difficult of conception when you recollect that circumstances analogous to it are extremely familiar, and of almost constant occurrence. There is not much more difficulty in conceiving the formation of a living worm within the body, than there is of conceiving the organization of a portion of lymph thrown out upon the surface of a serous membrane. What occurs in both cases is, that under the influence of the vital principle of the original animal, a portion of matter previously inorganic, assumes the properties of life, presents distinct traces of organization, vascularity, and sensibility. The only difference between them is, that in one case the organized mass remains adherent to the matrix, and in the other it is cast off, and forms a separate being. In the present state of our knowledge all speculation on the mechanism of the formation of worms must of necessity be nothing more than mere hypothesis. The idea which Bremser entertains on this subject is, that *intestinal worms* are formed by the presence of semi-assimilated nutritious matter in the digestive tube. Food taken into the system under ordinary circumstances, is converted into a substance fitted for the purposes of absorption and nutrition, but when the process is not perfected, it is not taken up by the absorbents, and is then, according to Bremser, converted into an animal substance. This appears to be but a crude idea, unsupported by any facts; and it would be more philosophical to say that we know nothing about the matter. Besides, worms occur in various parts of the body, as well as the digestive tube; and to suppose the presence of unassimilated matter in such situations would be only supposing an absurdity. Bremser brings forward, in support of this theory, that worms are of very frequent occurrence in cases where the assimilating powers are weak or deranged, and says that nothing is more common than to meet with an abundance of these animals in scrofulous persons, in those who have great appetites and bad digestion, and in children laboring under disease of the mesenteric glands. On the other hand, there are abundant instances of worms existing without the slightest apparent injury to the general health. In certain countries almost all the inhabitants have worms. But I believe all that we can affirm on this subject is this,—that they are not introduced from without, and that they are formed within the body by a process, the nature of which is exceedingly obscured.—*London Med. and Surg. Jour. May*, 1834. —*Ibid.*

8. *Pathology of Intestinal Worms.* By Wm. Stokes, M. D.—Can we connect the formation of intestinal worms with any known pathological condition of the intestinal tube? This is a question of no ordinary importance, for if we were able to connect their formation with an inflammatory or any other state of the digestive tube, it would furnish us with a key to correct and successful treatment. The school of Broussais are of opinion that worms are the result of an acute or chronic inflammation of the gastro-intestinal surface. This doctrine is by no means supported by the evidence of facts, for it has been established *that worms are found to exist not only in connection with every possible pathological condition of the intestinal canal, but also where the tube presented the appearance of perfect health.* We cannot, then, safely affirm that intestinal worms are connected with an inflammatory or non-inflammatory condition of the digestive tube. Andral states that he has found them in all conditions of the intestine, whether red or pale, dry, or covered with mucus. They are most commonly, he says, enveloped in a quantity of mucus, and there is some redness in the place where they are lodged, but this appears to be rather the effect of their presence than the cause. I believe it to be the fact, that persons in excellent health, and with the intestinal canal in the normal state, may have worms. Dogs who are killed while in a state of apparently perfect health, are often found to have a large quantity of tape-worm in their intestines. It is idle and hypothetic to say, that the formation of worms depends upon an inflammatory or non-inflammatory, an asthenic or sthenic condition of the digestive tube; their formation is owing to some modification of the vital power, the nature of which is unknown. I again repeat, that nothing can be stronger against the supposition that worms depend upon inflammation, than the fact of their being observed in considerable quantities in healthy individuals.—*Ibid.* —*Ibid.*

9. *On the Supposed Power of Worms of Perforating the Intestines.* By Wm. Stokes, M. D.—This question, which is an interesting one in many points of view, has been lately the subject of medico-legal discussion, and therefore demands a share of our attention. Of the different kinds of intestinal worms, the only one which is supposed to be capable of perforating the coats of the digestive tube, and escaping into the peritoneum or some adjoining organ, is the lumbricus, which is remarkable for its vigor and for the sharp and pointed shape of its head and tail. Many of the most eminent pathologists of modern times, and among the rest Andral, Rudolphi, and Carswell, are of opinion that these worms are totally incapable of perforating the intestinal tunics. Andral states, that there is no well-authenticated instance of this occurrence on record; and Rudolphi declares that they have no apparatus for effecting a passage through any continuous tissue. On the other side of the question, however, there are some curious facts and cases given, which, supposing the worms incapable of perforating, are very difficult to explain. Dr. Fischer, of Vienna, gives the case of a female, in whom the following circumstances were observed on dissection. Two circular orifices were found in the colon, communicating with the cavity of the peritoneum; in one of these openings a worm was discovered, one-half of which lay in the peritoneal sac, and the other in the intestine. No other worms were found in the digestive tube, but a second worm like the former was found in the peritoneum. Here we have a very remarkable coincidence of perforation of a portion of the gut, with the existence of one worm in the cavity of the peritoneum, and another of a similar description, as it would appear, in the act of making its way in the same direction. These circumstances, together with the existence of a double perforation, seem to be in favor of the idea, that the openings had been made by the corresponding worms. Another case is mentioned in the Elements of Pathological Anatomy, by Andral, and he quotes the case, not as one of perforation merely, but to show that the symptoms of effusion of matter into the peritoneum may, under certain circumstances, be nearly latent. The subject of this case, a young man laboring under phthisis, had a tumor near the umbilicus, which increased rapidly in size, and presented a distinct fluctuation. Soon afterwards the integuments gave way, and a large quantity of matter was discharged together with a lumbricus. During the progress of this disease there was some tympanitis, but little or no pain had been complained of. On dissection, there was a considerable number of worms, and a quantity of matter found in the peritoneum, and the perforation in the arch of the colon, corresponding with the extravasated matter. Bremser gives a very curious instance of this kind as occurring in a species of fish. In this case the fish died, and it would appear, says Bremser, that the worm finding some extraordinary change had taken place, was determined to take a peep and see what was the matter, for it had perforated not only the intestinal tube, but actually made a passage for itself through the whole body of the fish until it reached the water in which it had been lying. Here, finding that its world extended no further, it stopped, and began to make its way back again to its original situation by a new opening, so that when it was observed by Bremser the two ends were in the intestinal tube of the fish, and the middle portion external. This, however, does not resolve the question, as to whether lumbrici are capable of perforating the intestinal canal or not. My own impression on the subject is, that we have not as yet any distinct and unquestionable evidence of these worms being possessed of any perforating power; but it is a fact, that there are a great many cases on record of worms being discharged in considerable quantities from openings in the intestinal tube, and where it would appear that the openings had been formed, not so much by the action of the worms themselves as in consequence of their exciting an irritation in some portion of the intestine, followed by inflammation, ulceration, and escape of the contents of the tube into the peritoneum. There are many instances of this kind. An interesting case is mentioned of a female who was attacked with pain in the groin, followed by the appearance of a tumor, which she was directed to poultice by her medical attendant. After some time the integuments gave way, a quantity of matter was discharged, followed by a large lumbricus, and during the progress of the case, about one hundred of these animals were discharged through the opening. This is a well-authenticated case. Another case is mentioned of a patient who had been subject to constipation and violent attacks of colic. A tumor began to appear in the right hypochondrium, followed by pointing and ulceration of the integuments and a discharge of matter. A number of worms (I believe twenty-four) were discharged through the opening, which remained pervious, and the patient lived for many years afterwards with an artificial anus. This case appears to be not an example of direct perforation from worms, but of the accumulation of a mass of these animals in a particular portion of the intestine, giving rise to irritation, which terminates in ulcerative absorption of its tunics and escape of its contents. Inflammation is set up in some part of the intestine, this goes on until the coats are all destroyed, and the matter and worms escape into the peritoneal cavity; but if adhesion should prevent this, an opening will be formed in some part of the integuments covering the belly. In both cases the opening is produced, not by an exertion of the worms, but by an ulcerative and vital process. In support of this view, it has been observed, that worms have come out through these apertures not head foremost; the centre portion appears first, and you can draw it out like a loop. Such cases as the foregoing, then, cannot be fairly given as cases of perforation from worms, but as cases in which these animals, acting somewhat like foreign bodies, produced irritation, inflammation, and ulcerative absorption. There is a very curious case on record, of a patient laboring under abscess of the liver, which burst externally, and a lumbricus was discharged with the matter. The patient died; and on dissection it was found that the cavity of the abscess had a communication with the stomach, through which it was conceived that the lumbricus had got into the liver.—*Ibid.*—*Ibid.*

10. *Worms which inhabit the Intestinal Canal in Man.* By Wm. Stokes, M. D.—The worms which inhabit the intestinal canal in man are the following:—first, the lumbricus, or common round worm; next, we have the tape-worm, of which two varieties have been described; thirdly, we have the very curious worm, of which there is a specimen before me,—it inhabits the cœcum, and is called tricocephalus; lastly, we have the thread-worm, to which the name of oxyuris vermicularis has been lately given. The lumbricus generally inhabits some portion of the small intestine, but is also frequently found in the stomach. Persons have often vomited them, and they have been known to have crept out by the mouth. They have been found also in the pharynx, œsophagus, and large intestine. There is an interesting case mentioned by Andral of a child, who, in a state of apparently good health, was suddenly seized with symptoms of suffocation, and died. On dissection, it was found that a large lumbricus, which had come up from the stomach, had, when it arrived at the glottis, turned into its orifice, and by irritating the larynx, produced spasmodic closure of that organ, and suffocation.

The lumbricus presents very marked appearances of an advanced state of development. The male has a peculiarly formed penis, the female has her generative organs well developed, and both have an extensive alimentary canal. The tricocephalus is about an inch in length, terminating in a point; the sexes are different, and the male is distinguished from the female by the circular whirl of his tail,—it is always found in the cœcum. The small thread-worms, with which you are all acquainted, are almost exclusively found in the rectum. These worms are found in vast numbers in some children; and it is said that the quantities of them which are discharged by the West India negroes are extraordinary.

The tenia, or tape-worm, is generally found in the small intestine; but it has also been observed in the stomach, colon, and rectum. The length to which this animal sometimes attains is almost incredible. Bremser mentions a case in which a tape-worm 150 feet in length was discharged by stool. Another case is given, in which the tenia had the enormous length of 300 feet. I have myself seen a large wash-hand basin filled by a mass of tape-worm, discharged after a strong dose of castor oil and turpentine. Still more extraordinary instances are recorded. Thus, in the Copenhagen Transactions we read of a tape-worm 800 ells in length. But in all probability there has been an error in these measurements, and many worms have been taken for one. This is rendered probable by the fact observed by Robinus, who found in the body of a man who had before death discharged fragments of tape-worm, a tape-worm extending from the pylorus to within six inches of the anus. The length of this single worm was scarcely 30 feet. One interesting circumstance connected with this animal is, that it is inferior in its organization to every other species of worm. It appears to be merely a simple homogeneous, cellulo-gelatinous mass, without any division of sexes, and without a nervous system, or generative organs. It is said also to occur principally in persons whose powers of life are low; and if this be the case, as I believe it is in many instances, it furnishes us with a very curious and interesting fact. The other better developed kinds are found in persons of healthy, good constitutions; but the tape-worms, though sometimes met with in such persons, are generally found to occur in persons of low and weak diathesis. Here we see a curious connection between the product and the producing cause. —*Ibid.*—*Ibid.*

11. *Symptoms of Intestinal Worms.* By William Stokes, M. D.—It is a singular fact that we have not one single pathognomic sign of the existence of intestinal worms, except the circumstance of their being occasionally passed by stool, or vomited; almost all their symptoms are referable to irritation of the gastro-intestinal surface, and its sympathetic relations. Persons who are much subject to worms in these countries, are generally of a pale complexion, with a bluish circle round the eyes: the belly is more or less prominent, and there are various signs of irritation of the digestive tube, with itching at the nose and anus; head-ache; foul breath and tongue; irregular and sometimes canine appetite, nausea, hiccup, borborygmi, tenesmus, diarrhœa, and constipation. Though the patients take abundance of nutriment, they are generally thin and pale, and in such cases there is either one or two very large worms, or a great number of smaller ones, or their presence is complicated with disease of the intestinal canal. Such persons are also observed to be of an indolent and languid habit; they have perspirations, disturbed sleep, with grinding of the teeth, and irregularity of pulse.

The sympathetic irritations, produced by worms, are numerous and extraordinary. The genital organs may be excited, and we may have priapism and seminal emissions in the male, and irritation amounting to nymphomania in the female. There is a very singular case on record of a female, aged 70, being seized with a violent attack of nymphomania from this cause. The nervous affections, produced by worms, are so Protean and so numerous, that it would be almost impossible to detail them; in fact, there is not a single nervous disorder which may not be simulated by the sympathetic irritation of worms. Epilepsy, hysteria, convulsions, dilatation of the pupil, amaurosis, symptoms of hydrocephalus, and even mania are among the affections of the nervous centres, or their immediate connections, which, in repeated instances, have been found to depend on the presence of worms. Kraus gives an extraordinary case of a man, who, at a very advanced age, became subject, from this cause, to fits of continued and inordinate laughter.

There is another case on record of convulsions depending on worms, which, like those from the bite of the tarantula, are said to have been soothed and relieved by music. Hufeland, in his journal, mentions a case of yellow vision from the same cause, and there are several instances of aphonia and mania on record, which have yielded to treatment which had removed intestinal worms. A case is mentioned of a person who got violent spasmodic action of the muscles of the eye, producing inversion of that organ to such a degree, that the eyeball appeared to be nothing more than a mass of red flesh. A case is recorded by Serres, in which the symptoms strongly resembled those of hydrophobia, and it is probable that some of the cases of hydrophobia, said to have been treated successfully, were nothing more than this extraordinary irritation of the nervous system produced by worms. I saw myself a case, in which two eminent physicians made the diagnosis of hydrocephalus; it was that of a child who was certainly to all appearance laboring under cerebral disease, for he had convulsions, coma, and dilated pupils. It was remarkable, however, in this case, that the treatment directed to the head, though early and well applied, proved totally inefficacious. A large dose of calomel was given, and some lumbrici passed; in the space of two or three hours there was an evident improvement, and the child quickly recovered.

During the course of practice I have met with several examples of affections of the respiratory organs, depending upon the irritation of worms. This affection has been long known. I recollect the case of a boy who was brought to me with an extraordinary affection of the chest. He was of a gross habit of body, of a flabby scrofulous appearance, and laboring under disease of the elbow-joint; but his chief complaint was, that he passed the night in great distress from incessant cough and wheezing. On examining the chest, I found the respiration healthy, and no other symptom of pulmonary derangement except very slight bronchitic rale. On expressing my opinion of the case to the mother, she said that he was easy during the day, but that his condition was very different at night. To ascertain the truth, I took the child into the hospital, and found that her statement was substantially correct; for from four o'clock in the afternoon until next morning, he was in a state of orthopnœa, with loud, ringing, incessant cough. During the rest of the day he was free from cough, and tolerably quiet. The case was treated with calomel and ipecacuanha, tartar emetic and other similar remedies, but the disease was rather exasperated than improved. The boy had swelled belly and constipation, and for this he was ordered to take a dose of turpentine and castor oil. He passed some worms with relief to the existing symptoms, and from the consideration of this, and the failure of the treatment for bronchitis, we were determined to persevere in the use of anthelmintic medicines, and, for this purpose, put the child on syrup of cowhage, to be followed by castor oil draughts. He passed vast quantities of thread-worms in the course of a few days, and when they had been all removed, the cough disappeared altogether, but as long as any of them remained, the symptoms of pulmonary irritation continued. There could be no doubt that this was a case of intermittent bronchial irritation from worms, for their evacuation was immediately followed by a complete cessation of cough and dyspnœa. I have also, since the foregoing, met with many other instances of a similar description. A young girl came into the Meath Hospital with chronic bronchitis and some degree of hepatization at the lower part of the left lung. Having heard from her friends that she was extremely subject to worms, I determined to try what would result from the use of anthelmintic medicines, and put her on the syrup of cowhage with aloetic pills. Under this treatment the cough was quickly removed, and the lower portion of the lung recovered its permeability. Here, it was remarkable, that not only irritation of the bronchial mucus membrane, but even solidification of the lung, were cured by treatment calculated to remove worms. Mr. Ramsay, in his paper published in the Medico-Chirurgical Transactions, gives several cases of hæmoptysis from this cause. I think I have seen several cases of phthisis, where the original source of pulmonary irritation seemed to be the existence of intestinal worms.

Let me here, however, remind you, that we should be cautious in attributing too much to worms as the causes of morbid symptoms. There are several reasons why you should be on your guard in this respect, one of the most obvious of which is this,—it does not follow, in the first place, that the symptoms in any particular case are produced by worms, because the same cause, which may have predisposed to the formation of worms, may have produced the symptoms in question, and there may be merely a coincidence of worms and of these symptoms. Even if we look to the results of treatment, there is a great deal of doubt and difficulty. There are many cases on record which are described as cases of epilepsy from worms, and where all the symptoms have subsided under the use of anthelmintic medicines. In many of these cases we find the medicine chiefly employed has been oil of turpentine, and I need not tell you, that this is an excellent remedy in many cases of epilepsy totally uncomplicated with worms. The results of such cases do not necessarily prove that worms were the source of irritation. Again, immense injury is frequently done to children in persisting in the anthelmintic treatment for the supposed existence of worms. Recollect, the prominent phenomena of worms in the intestines are irritations of the digestive system and of other functions. Now, it is very well known that these symptoms may occur with or without worms. If, then, you have a case where these phenomena are present without the coexistence of worms, and if, under a mistaken impression, you treat it with anthelmintic medicines, you inflict a double injury; you exasperate the original disease by the drastic and irritating medicines which are ordinarily used for the removal of worms, and you do an indirect injury by neglecting to adopt proper means of treatment. There is nothing more common than to see children laboring under some irritation of the digestive tube, which is mistaken for worms, purged again and again until they get incurable enteritis or tabes mesenterica. When a child has foul tongue and breath, picking of the nose, diarrhœa, and turbid urine, it is a common notion that he is laboring under worms. If he gets feverish, it is said to be the worm fever, and the anthelmintic treatment is pursued with unabated vigor. Now, I believe that a great majority of such cases are in reality disease of the mucous surface of the intestine, and that the consequent feverishness is dependent on this state. Another reason why you should be cautious is this,—in persons of an hypochondriac habit, there is nothing more injurious than their getting the idea that they have a worm in their bowels. When once this notion gets into the head of an hypochondriac, it is generally impossible to eradicate it. Some of the most melancholy and fixed cases of hypochondriacism are produced in this way; every symptom is attributed to the worm, the patient is in a state of constant feverish anxiety about it, he talks of nothing else, and is constantly taking medicines to expel it, to the great detriment of his general health, and with a manifest exacerbation of his symptoms. Medical men should be extremely cautious on this point. The patient is perhaps a female of hypochondriac and nervous habit; she has gnawing sensations about the epigastrium, which she supposes to depend upon the presence of a worm, and an injudicious practitioner favors the notion. He gives her various medicines to expel the worm; no worm is passed; she becomes more anxious, takes more medicine, and gets weak and emaciated. She then begins to think that all the nutritious matter in her body is going to support the worm, falls into a desponding state, and continues for the rest of her life an incurable hypochondriac.—*Ibid, May* 10, 1834.—*Ibid.*

13. *Causes of Worms.* By William Stokes, M. D.—Our knowledge is very scanty and inaccurate respecting the exciting causes of worms. The following, however, are generally looked upon as remote causes:—foul air, residence in damp and unhealthy situations, sedentary habits, and want of wholesome exercise, overfeeding, the constant use of certain articles of diet, as farinaceous substances, milk, cheese, sugar, etc. An eminent authority, (Bremser,) asserts, as I have already stated, that unabsorbed chyle in the digestive tube constitutes the most fertile source of worms. It is a common idea, that poor diet has a strong tendency to give rise to the formation of these animals, but it has been frequently observed that worms are met with in persons who are by no means in want of nourishment; and it is said that in cases where nutrition has been diminished in man and other animals, the worms die. If this be the case, it would appear that, so far from being the exciting causes of worms, poor diet rather tends to favor their removal. Uncooked vegetables and fruits are also reckoned among the causes of worms, but I believe this arises from the mistaken notion that the ova of intestinal worms occur in vegetables, and, being taken with them into the stomach, are there developed, or even changed in their organization, a position which we have already proved to have no foundation in truth. Persons who live principally on vegetable food have not been observed to labor under worms in a comparatively greater degree than those who use an animal diet. It is said that the Swiss, who consume a great deal of vegetables, are very subject to worms, but other nations who live in a similar way have not been remarkable for the same liability.

Worms have been stated to be occasionally epidemic. It is not very easy to determine this point, but it has been remarked, that at particular periods these animals have been more than usually frequent and numerous. Many authors have described an epidemic of what has been called *verminous fever*, that is to say, fever of a gastric or bilious character, accompanied by worms in quantity. It is hard to say what the nature of this fever really was, and whether it might or might not be fever with irritation of the digestive apparatus, one of the consequences of which was a discharge of worms already existing. That worms are endemic, is a proposition very easily conceived, for we see it illustrated by the extraordinary prevalence of these animals in sheep which are kept in low, damp pastures. In such situations worms are met with in great abundance in the liver and other parts of these animals.

It would appear, from the following remarkable case, detailed by Bremser, that the use of milk and farinaceous food predisposes to the formation of intestinal worms. This gentleman, who was physician to a monastery, and had ample opportunity of studying the habits of its inmates, was called to visit one of the oldest of the monks, who was said to be laboring under great derangement of the digestive system. On inquiry, he found that the patient had lived for sixty years in excellent health, using animal food, which, however, he had been latterly induced to change for farinaceous diet and milk. For a few days this agreed tolerably well with him, and then he began to be tormented with colicky pains, flatulence, sour eructations, and other distressing symptoms. His physician gave him some purgative medicine, and he passed a large quantity of tape-worm with relief; the treatment was persevered in, his former mode of living was resumed, and he recovered quickly. This case bears strongly against the fanciful hypothesis that the ova of worms are transmitted in the act of generation; for how could it be possible that the ovum of this tape-worm, transmitted in this manner, could remain undeveloped in the system for the space of sixty years! This case derives additional interest from the fact of a change to a farinaceous diet being apparently connected with the formation of worms.

Another remarkable case is given by the same author. The patient was a married female who had twelve children,—six boys and six girls. This woman observed, that whenever she was pregnant of a girl, she had a greater longing for milk and farinaceous food, and lived on those articles of diet almost exclusively. After living in this way for some time, she uniformly got an attack of worms, and this, as well as the longing for vegetables, coincided with the birth of a female child so invariably, that she was able to tell with certainty whether the child she carried was a male or a female. This is a singular and well authenticated fact.—*Ibid.*—*Ibid.*

13. *Treatment of Worms.* By Wm. Stokes, M. D.—The treatment of worms is, generally speaking, extremely simple, the principles of treatment in the various kinds of intestinal worms being nearly the same. Simple as they are, however, some persons entertain false notions respecting them. They appear to think that all they have to do is to evacuate the worms; and, having accomplished this, they rest satisfied, and take no steps to prevent their recurrence. But the mere evacuation of worms is no proof of a cure; to effect this you must prevent their return. From what you have learned with respect to their exciting causes, you will be able to give such directions as to the patient's mode of living as will obviate their recurrence; and, with regard to the means to be adopted for removing them, we may divide them into the following:—We have, in the first place what is called the mechanical treatment, next the specific, and, lastly, the purgative treatment. The first and last are nearly connected. For instance, purgatives appear to act in the same way as mechanical anthelmintics, by irritating the mucous surface of the intestine and the worm, and thus causing its dislodgment and expulsion.

Among the principal mechanical anthelmintics are filings of tin, cowhage, powdered charcoal, and crude mercury. Among the specific are a variety of substances, most of which have a strong and peculiar smell. This is a very curious fact. Valerian, assafœtida, camphor, ether, and other odorous substances have been found to be anthelmintic, and the Geoffræa inermis, which has been employed for this purpose, is remarkable for its strong unpleasant odor. The same thing may be said of tobacco, the oil of chenopodium or wormseed, garlic, artemisia absinthium, and many others. With respect to purgatives, there is not one in the whole list, particularly those of the drastic kind, which may not be looked upon as an anthelmintic.

It is the opinion of the most eminent men, that the thread-worm is the most difficult to expel, because they are generated with an extraordinary rapidity, and accumulate in a very short space of time. You are satisfied of their existence, have seen them in the alvine discharges, and the patient has all the ordinary symptoms. Well, what is the best way of getting rid of them? You shall commence by the exhibition of a mercurial. It is difficult to explain why it is that mercury has such an effect in removing these worms, but the experience of the best practitioners can be adduced in proof of its efficacy. The statements of Dr. Latham, of London, and of many practitioners in this country and on the continent, go to prove this. In whatever way it acts, mercury appears to be a powerful anthelmintic; and it is a fact that these worms have been expelled where it was given in very small doses, and not sufficient to operate as a purgative. The best plan is, first to give a mercurial purgative, and then to have recourse to the mechanical treatment, giving with this view, the syrup of cowhage, one of the most efficacious of this class. It is a remedy which is easily managed, and will do no harm; for, though it produces violent itching when applied to the cutaneous surface, it produces very little sensible effect on the intestinal mucous membrane. The form which I employ is the following:—Take of the hairs of the dolichos pruriens one scruple, syrup of orange-peel an ounce; of this an electuary or syrup is to be made, of which you may give a child a tea-spoonful three times a day. This is the remedy on which the West India practitioners, who have frequently to treat this affection in the negroes, place the greatest reliance; and you will find that if you employ it, a vast number of worms will be often passed. It should be continued for two or three days, and then a purgative must be given, after the operation of which it may be again resumed, if necessary. An excellent adjuvant to this is the use of aloetic injections, composed of two parts of milk, and one of the decoction of aloes. In this way you will be able to remove a vast quantity of these little animals from the rectum. It has also been observed, that injections of cold, fresh or salt water have a great power in promoting their expulsion. Bremser mentions that in cases where these worms pass from the rectum into the vagina in females, and excite irritation, there is nothing so effectual in destroying them as injections of cold water and vinegar. This you should bear in mind. You should also remember, in the case of administration of syrup of cowhage, to give strict orders not to let any of it drop on the child's skin, as it would excite a great deal of irritation. You should forewarn the attendants of its effects on the skin; and if any of it should be spilled on the hands, neck, or face, the best thing is to wipe and wash the part well, and then rub it with a little almond-oil.

For the expulsion of lumbrici there is nothing so successful as the ordinary purgative treatment. A bolus, composed of calomel, rhubarb, and jalap, will answer this purpose extremely well; you may also use the syrup of cowhage with much advantage. Bremser gives a formula for an electuary, which I have not tried, but have no doubt of its value, for it appears to combine all the qualities of a good vermifuge electuary. It is made as follows:—Take of the seeds of santonicum, and of the flowers and leaves of tansy, reduced to powder, each half an ounce. Here you have two anthelmintics of the specific kind. Add to these two drachms of powdered valerian: another. You then combine with these, two drachms of sulphate of potass, and a drachm and a half of jalap: these are purgatives. You then make them up into an electuary with syrup of squill, which is also an anthelmintic of the specific kind. Of this electuary two or three tea-spoonfuls are to be taken during the course of a day. Bremser states that this combination is of great value, particularly against lumbrici and tape-worm.

The treatment of tape-worm is not difficult. All the specific and mechanical anthelmintics are useful in promoting its expulsion, but there is nothing which appears to have such a powerful effect as full doses of turpentine and castor oil. This constitutes the best remedy we possess against the tænia; but if you wish to get rid of it entirely you must give the turpentine in full doses. You will frequently be astonished at the vast quantities of this worm which will be passed. When you give turpentine, it is safer to order a full dose of it, for if it be given in small quantities it is very apt to irritate the urinary organs. Half an ounce of turpentine, with the same quantity of castor oil, form an efficacious, though very disagreeable draught. You may, however, obviate its nauseousness by the addition of a small quantity of camphorated tincture of opium and mucilage of gum Arabic. The celebrated empyreumatic oil of Chabert is, in my mind, nothing more than a modification of the turpentine. This is the remedy which Bremser looks upon as most efficacious against the tape-worm. You have all, I presume, heard of the animal oil of Dippel—the oil which is produced by the distillation of bones or hartshorn shavings. To one part of this are added three parts of turpentine: these are left to combine for four days, and then distilled; the first three parts of oil which come over are called the empyreumatic oil of Chabert. It is an exceedingly nauseous remedy, has a most disgusting smell, and is seldom used in this country. Bremser recommends it to be taken in doses of a tea-spoonful three times a day. Some persons who have tried it have assured me that it is extremely difficult to be taken, and that it excites a train of most disagreeable abdominal sensations. Bremser, however, thinks highly of it; he is in the habit of directing his patients to take it for three or four successive days, then to omit for a day or two, and then to return to it again, and he says that it not only succeeds in evacuating the worm, but also in preventing its return. In addition to this, he recommends the use of a fortifying tincture, which I think very useful in worm cases. It is a combination of one of the salts of iron, with a preparation of aloes. If you take equal parts of the muriated tincture of iron and tincture of aloes you will have a remedy somewhat similar to the strengthening tincture of Bremser. Twenty drops of this mixture, taken three or four times a day, will prevent the recurrence of worms. —*Ibid, of May* 3, 1834.—*Ibid.*

14. *Diagnosis of Inflammation of the Spinal Dura Mater.* By Professor Albers, of Bonn.—From two cases observed by himself, as well as analogous cases related by other practitioners, Professor Albers is of opinion that inflammation of the spinal dura mater may be distinguished by characteristic symptoms from inflammation of the spinal arachnoid and spinal marrow, with which it has hitherto been confounded. These symptoms are, 1st, acute pain extending to the lower limbs and lower parts of the body; 2d, convulsive motions of different parts; 3d, tremors; 4th, difficulty of expelling urine and fæces; 5th, sensation of constriction around the body. The professor explains these different phenomena in the following manner:—

1. The *acute pain* in the parts situated below the seat of the inflammation was noticed in the two cases reported by the author, as well as in the first observations of M. Funk. This symptom has been repeatedly noticed in cases of tetanus. The pain is usually accompanied with a sense of pricking and tearing; and these distressing feelings are in most instances referred to the epigastrium, hips, and thighs. In very severe cases so acutely sensitive may be certain parts that even the slightest touch causes excruciating pain and cramps. Now this symptom, or rather chain of symptoms, is not observed when the archnoid and pia mater, or when the substance of the medulla itself is affected: under these circumstances, after the pain and convulsive movements have continued for some time, a paralytic weakness usually supervenes. On the contrary, paralysis is certainly not a common result or effect of inflammation of the dura mater, unless the disease has involved the parts situated within its sheath.

2. *Convulsive movements.*—When the cervical portion of the spinal dura mater is affected, the muscles of the face and neck are usually drawn into irregular contractions, along with those of the chest and abdomen. The upper extremities are affected more tardily, and sometimes not at all, until a few days before death. It is not, however, unfrequent that severe and irregularly returning pains are felt from the shoulders down the arms; certainly the upper extremities are more generally affected than the lower, when the disease is situated in the cervical region. When the whole extent of the cervical section of the medulla is inflamed, the upper extremities are almost certainly affected; but when the inflammation is limited to its inferior portion, they may escape altogether, or exhibit only a trembling movement or agitation, while the lower limbs are all the time contracted with tetanic spasms. Sometimes the arms are spasmodically affected, while the thighs and legs are paralysed. Under these circumstances, we may expect to find on dissection an effusion of reddish serum within the sheath of the dura mater, at its lower part. As long as the quantity of this serum is small, it may act as an irritant on the cauda equina, and may thus cause convulsions of the inferior extremities; but when it becomes more abundant, it induces, in consequence of the compression, partial or total paralysis.

Professor Albers remarks, that the tetanic convulsions which attend inflammation of the dura mater are almost always of the "tonic," and very rarely of the "clonic" kind. The cause of the persistence and intensity of the spasm is no doubt attributable to the irritation which the diseased envelope exercises on the contained medulla, being constant and not remitting. "Now, this is not the case when the medulla itself or its immediate coverings are affected. The state of high excitement lasts but for a short time; hence the violent convulsions are either from the exhaustion of the nervous energy, or from the compression on the nervous mass, speedily followed by paralytic weakness. On the other hand, this state of paralysis seldom occurs in inflammation of the dura mater, except for a few hours before death."

3. *Tremulous agitation of different parts of the body.*—This symptom is usually most conspicuous in the early stages of the disease, when the patient attempts to walk. He finds that he cannot keep his head steady for a moment, and that his arms, body, and legs are in a constant tremor. This is one sort of the "paralysis tremens" of Dr. Cooke. It may be observed, that the involuntary action of the muscles we are now alluding to, always ceases when the patient is in the horizontal position.. As the disease lasts, this symptom becomes less and less distinctly marked, and either disappears altogether, when a favorable termination may be anticipated, or it is succeeded by a tetanic stiffness or contraction of the limbs. As soon as tetanus begins to appear, the tremulous agitation ceases.

4. *Difficulty in expelling the urinary and alvine evacuations.*—In general, the paralytic affection of the bladder precedes that of the rectum; and, indeed, the latter viscus is sometimes but little affected till within a short time before death. The true nature of the urinary local distress is not unfrequently mistaken at first, and it is only when the bladder has become enormously distended, that the practitioner is made aware of the existing evil.

5. *The feeling of constriction round the body.*—When the cervical portion of the medulla is inflamed, the sense of constriction is usually experienced round the lower part of the thorax, extending from the dorsal vertebræ to the epigastric region. When the lower part of the dorsal, or when the lumber division is the seat of the disease, this symptom is remarkable chiefly in a line, running almost parallel to the anterior and superior crista of the os ilii. The sense of constriction is not unfrequently accompanied with positive pain; it is always an unfavorable sign, as it indicates the intensity of the inflammatory action. When once established, it is, even under the most fortunate circumstances, very obstinate of removal.

In concluding these remarks on inflammation of the spinal dura mater, we may state that the functions and faculties of the brain may remain uninjured, under the most serious disease of the medulla spinalis. —*Archiv. Generales, Sept.* 1834, *from Græfe and Walter's Journal, XIX.* 3.—*Ibid.*

15. *Of the Influence of Professions upon Phthisis Pulmonalis.* By M. Lombard, of Geneva.—In the list of the professions which dispose to phthisis, all are enumerated with the exception of three or four, and these have been noticed as preventing the disease, viz: butchers, coal miners, fishmongers, and tanners. The author of this memoir, M. Lombard, of Geneva, judges that this list might be greatly enlarged. At first, by analyzing the bills of mortality collected in the hospitals of several large cities, and the observations collected in the official records of Geneva, he classes the professions into two series, according as they number more or less than 114 phthisical cases in 1000 deaths. After many calculations, intended to expose apparent contradictions, furnished by the statistics of different countriès, and of the various trades, he concludes that the following professions have been improperly classed among those which dispose to phthisis. On the contrary, they appear rather to be opposed to its development: lawyers, dyers, founders, stocking-makers, bleachers, bakers, quarry-men, chandlers, brasiers, gilders, blacksmiths, miners, porters, locksmiths, and wharf-porters. Passing to examination of the causes which produce these results, M. Lombard at first notices the state of ease or poverty which the different professions imply or suppose, and he concludes that the poorer classes are ten times more accessible to phthisis than those who are situated in ease and comfort. He then passes to muscular exercises. A sedentary life causes a much greater number of cases of phthisis than an active life. The sedentary professions give as a mean number of phthisical cases, 141 in 1000 deaths. Whereas the active professions, in the same number of deaths, only give 89 cases of phthisis. The exercise of the voice is not, notwithstanding the general opinion, a circumstance which assists the development of phthisis. If there are some persons who experience the bad effects of the constant exercise of the voice, this exercise is, in general more advantageous than injurious. The professions which exercise the voice, only afford 75 cases of phthisis in 1000 deaths. With regard to the bent position, it is necessary to distinguish the trades which force this position whilst great muscular exercise is employed: hatters, tanners, gardners, wood-sawyers, and the trades which in bending the body leave it in almost complete repose: tailors, engravers, watch-makers, shoe-makers. The latter afford a mean of 134 cases of phthisis in 1000 deaths; the former only 83. Hence, although they might induce us to consider the constraint occasioned in the functions of the lungs, as a frequent cause of phthisis, this circumstance appears to exert but a secondary influence, and is more than counterbalanced by constant muscular exercise.

Influence of the purity or impurity of the atmosphere. In the professions exercised in the open air, 75 cases of phthisis in 1000 deaths; in professions exercised in factories, 138. When the factories are large and open, phthisis is less frequent than in small and close factories. The healthfulness of the atmosphere which surrounds factories is often altered by foreign particles, the contact of which with the lungs have a remarkable influence upon the development of phthisis. Foreign particles may be dissolved in the atmosphere or simply suspended in the air. Aqueous vapors. All professions which are exercised in the midst of these vapors, as tanners, bleachers, water-men, water-carriers, laundresses, are classed below the mean, 114. The uniformity of this result is as remarkable, as the theory would lead us to a contrary opinion. Observation shows, that cold and moist climates are those in which phthisis exercises its greatest ravages. And yet the occupations in which the workmen which are surrounded by watery emanations only give 53 phthisical cases in 1000 deaths. This conclusion, drawn from observations at Geneva, is opposed to that which M. Benoiston de Chatauneuf has drawn from observations at Paris. According to M. Lombard, the bleachers of Paris are exposed to many causes of phthisis independent of mere moisture of the atmosphere. A dry and warm air appears to be an active cause of phthisis pulmonalis. The proportion of phthisical cases in 1000 deaths, of iron-mongers, jewellers, makers of files, founders, forgers, is about 127. The frequency of phthisis with other workmen, as watch-makers, toy-men, mounters of watch-cases, goldsmiths, depends, without doubt, upon the high temperature of the stoves which they use, a temperature which dries and purifies the air of their workshops. The influence of animal emanation, appears to be as advantageous as that of a moist atmosphere, among butchers, tanners, chandlers, nurses of the sick, butcher's wives. The proportion of phthisical cases in 1000 deaths is only 60. An atmosphere which is charged with the emanations of living plants may pass as a preservative from phthisis; gardners are less subject to it than farmers; in 100 deaths of gardners only 4 were owing to phthisis. This is not the fact with regard to emanations from dead or fermenting vegetable matter: attendants on wine cellars and bakers are very subject to phthisis. The number of phthisical cases among starch-makers is less than the mean. Among varnishers the proportion of phthisical cases is considerable: in 65 deaths of varnishers of paintings at Geneva, 24 succumbed to phthisis. Picture painters, although exposed to emanations of turpentine and the drying oils, succumb less frequently than varnishers, because they are not obliged, as these latter, to saturate themselves, as it were, with noxious emanations, by closing so carefully their work shops in order to prevent the dust from attaching itself to the varnish. Emanations from the mineral acids do not appear to be deleterious. The nitric acid is used by hatters, gilders, assayers, goldsmiths. Of these four occupations but one is above the mean, 114, the rest count very few phthisical cases. Phthisis is also rare with those who are engaged in making chlorine. As to the metallic emanations, the statistics of Geneva are opposed to those of Paris, collected by M. Benoiston; and M. Lombard suspends his opinion until there is more ample information. There is the same uncertainty as regards the vapors from lead, arsenic, antimony, and copper; at the same time he is inclined to think that these vapors are less prejudicial than is commonly supposed. Those occupations which expose the workmen to an atmosphere charged with foreign particles, may be divided into two classes, according as the molecules are large or very much divided. In the first class the proportion of phthisical cases in 1000 deaths is about 137, in the second about 152. The mineral dusts cause 177 in 1000 deaths; vegetable, 105; animal, 144. Then, in fact, the most injurious of all foreign bodies which can exist in the atmosphere is the impalpable dust of very hard substances. Moreover, the mineral dusts are the most deleterious, particularly those of steel and silica. Dr. Knight, of Sheffield, has remarked, that there is not, at that place, a single polisher of steel forks who has attained his thirty-first year. This shortness of life is remarkable with the cutters of flints, freestone, and crystals. The filamentous dust of cotton, wool, feathers, etc. is more injurious than that of flour or starch. We will now recapitulate the professions in order as they exert an injurious or preservative influence. Mineral and vegetable emanations, 176 cases of phthisis in 1000 deaths; various dusts,

145; sedentary life, 140; a life passed in manufactories, 138; warm and dry air, 127; bent postures, 122; movements of the arms causing shocks to the chest, 116. Let us recollect that the mean of phthisical cases, among all kinds of diseases, is 114 in 1000 deaths. Now let us enumerate those that have a preservative effect: an active life, with muscular exercise, 89 cases of phthisis in 1000 deaths; exercise of the voice, 75; a life passed in the open air, 73; animal emanations, 60; aqueous vapors, 53. M. Lombard devotes the latter part of his paper to the therapeutic applications, which there is no necessity of repeating here, they may be induced very naturally from the facts detailed. We will only note the kind of climate which he advises physicians to recommend to their phthisical patients. This climate ought to be moist, at the same time warm and serene. He also prefers Rome and Pisa to Nice, to Naples, and particularly to the dry and brisk climate of Marseilles and of Montpelier.—*Gaz. Med. May* 25*th*, 1834, *from Annales d'Hygiene et de Med. Leg. Jan.* 1834.—*Ibid.*

16. *On the Probable Duration of Life among Medical Practitioners.*—By Professor Casper, of Berlin. There is no part of medical statistics more painfully interesting, and at the same time important, than that which relates to the probabilities of the duration of life. Inquiry on the subject, it is true, scarcely tends to lift the thick veil which conceals the limits of individual life, and has little power to satisfy the pardonable curiosity which we all have, to know how long we may have to live: yet, when applied to masses, it enables us to arrive at results of great consequence, whenever provision is to be made for the duration of life in general, or for the probable duration of it at certain epochs.

For upwards of ten years I have been engaged, during my leisure moments, in researches of this kind—the difficulties of which can only be compensated by the value attaching to each result. I have considered most of the circumstances which tend to modify the duration of human life, and those more particularly which are connected with the practice of the different professions. Much has been attempted within the last two hundred years on this subject by mathematical and statistical enquirers, yet much remains to be done; for, in fact, with the exception of Deparciex's work on the mean duration of life among monastic persons of both sexes, we cannot be satisfied with any thing that has hitherto been accomplished.

Although I should not feel justified in laying at present the whole of my work before the public, there is, notwithstanding a portion of it with the correctness of which I am so well satisfied as not to hesitate in publishing it: the portion or fragment to which I allude, relates to the probabilities of the duration of life among medical men.

Establishments having a close connection with civilization, such as life insurance companies, those for annuities to survivors, for mutual insurance, etc. have become numerous of late years; and the institution, set on foot by the illustrious Hufeland, for the benefit of members of the profession, may serve as a model of the kind. But none of these establishments, as the experienced can testify, and as lamentable events have too clearly shown, can possibly subsist for any lengthened period, unless founded on very exact tables of mortality. It was with this impression that the following tabular view was formed.

In order to obtain the most satisfactory points of comparison, I have noted down 624 cases of death, occurring among physicians and surgeons, mostly all Germans,—and excepting anatomists, veterinarians, naturalists, and medical men, who are solely devoted to literature.* I have taken the age of twenty-three as my *point de depart*, both for the medical as well as the other professions, (a comparison of which it is my intention hereafter to make public;) but if others should choose twenty-four or twenty-five as the age from which we should begin to calculate, it in nowise interferes with the exactitude of the results.

My chief authorities in ascertaining the dates of the births and deaths of those on whom I reckoned, were Ersch, in his Manual of Literature, (Leipsic, 1822,) and Voigt, in his Necrology, (1833,) whose valuable collection is known to have been principally formed from manuscripts communicated by the friends and relatives of the deceased.

In column A of the following table we have the ages at which our medical men died: B contains the number of deaths at those ages.

Now we suppose the 624 individuals on whose deaths the table is constructed to have been contemporaries, or that they were all aged 23 at the same time. On this supposition the cypher 624 is placed in column C opposite the age of 23; and as there are two deaths for that age, 622 is the cypher which must stand next in column C, corresponding with the age of 24 in column A. Thus the column C is formed throughout, until the whole of the 624 are deceased, which is found to be at the 92d year. Column D exhibits the number of years which may be allotted to each period—that is, the probable duration of life according to Halley's method.† I have preferred the form which Halley follows to that of Deparcieux, as being less complicated, at the same time that it leads to the same results. By Halley's method, we find the probable duration of life very readily: we look into the column C to ascertain at what age only half those living at any given period survive. Thus, if of 122 medical men who are 72 years of age, we find that the half are dead at 77, it would seem that the chances of the 122 reaching that age, and of not reaching, it are equal; and the difference of the ages, (77—72)—nearly five years—is the length of time which those of 72 may hope to live.‡ Nothing positive, of course, can be predicated from the table respecting the probable duration of the life of any individual; but the general conclusions, as experience has proved, are nevertheless *extremely certain.*

---

*I have since calculated the mean duration of the lives of seventy-six other medical men—thus making up the entire number 700; but I have not thought it necessary to enlarge my table by the addition of the results, which only serve to confirm those already obtained.

†Philosophical Transactions for 1691 and 1693.

‡In general terms: if $m$ be the number of persons who have attained the age $a$, and that at the age $n$ the number is found to be reduced to $\frac{1}{2}m$, $n$ will express the probability of vital duration, and $n-a$ the number of years on which the individuals may calculate.

*Table of the Mortality of Medical Men.*

| A. Age. | B. Deaths. | C. Living. | D. Probable Life. | A. Age. | B. Deaths. | C. Living. | D. Probable Life. |
|---|---|---|---|---|---|---|---|
| Years. | | | | Years. | | | |
| 23 | 2 | 624 | 35, 5 | 58 | 10 | 317 | 11, 0 |
| 24 | 1 | 622 | 34, 4 | 59 | 17 | 307 | 10, 6 |
| 25 | 4 | 621 | 35, 4 | 60 | 12 | 290 | 10, 3 |
| 26 | 3 | 617 | 33, 0 | 61 | 15 | 278 | 9, 7 |
| 27 | 7 | 614 | 32, 0 | 62 | 14 | 263 | 9, 0 |
| 28 | 5 | 607 | 31, 4 | 63 | 19 | 249 | 8, 8 |
| 29 | 5 | 602 | 30, 1 | 64 | 20 | 230 | 8, 5 |
| 30 | 5 | 597 | 29, 8 | 65 | 11 | 210 | 8, 0 |
| 31 | 11 | 592 | 28, 6 | 66 | 18 | 199 | 7, 5 |
| 32 | 8 | 581 | 28, 0 | 67 | 6 | 181 | 7, 2 |
| 33 | 11 | 573 | 27, 6 | 68 | 16 | 175 | 6, 5 |
| 34 | 11 | 562 | 26, 8 | 69 | 9 | 159 | 6, 0 |
| 35 | 8 | 551 | 26, 0 | 70 | 17 | 150 | 5, 5 |
| 36 | 7 | 543 | 25, 5 | 71 | 11 | 133 | 5, 4 |
| 37 | 8 | 536 | 24, 7 | 72 | 15 | 122 | 5, 0 |
| 38 | 14 | 528 | 24, 0 | 73 | 14 | 107 | 5, 0 |
| 39 | 8 | 514 | 23, 5 | 74 | 13 | 93 | 4, 7 |
| 40 | 9 | 506 | 22, 7 | 75 | 10 | 80 | 4, 6 |
| 41 | 11 | 497 | 22, 0 | 76 | 9 | 70 | 4, 4 |
| 42 | 6 | 486 | 21, 3 | 77 | 8 | 61 | 3, 9 |
| 43 | 8 | 480 | 20, 3 | 78 | 10 | 53 | 3, 3 |
| 44 | 8 | 472 | 19, 7 | 79 | 4 | 43 | 3, 0 |
| 45 | 11 | 464 | 19, 0 | 80 | 11 | 39 | 3, 0 |
| 46 | 4 | 453 | 18, 2 | 81 | 6 | 28 | 4, 0 |
| 47 | 14 | 449 | 17, 3 | 82 | 3 | 22 | 4, 0 |
| 48 | 11 | 435 | 16, 7 | 83 | 3 | 19 | 4, 0 |
| 49 | 12 | 424 | 16, 0 | 84 | 2 | 16 | 4, 0 |
| 50 | 13 | 412 | 15, 4 | 85 | 3 | 14 | 4, 0 |
| 51 | 8 | 399 | 15, 0 | 86 | 2 | 11 | 3, 5 |
| 52 | 11 | 391 | 14, 2 | 87 | — | 9 | 2, 6 |
| 53 | 10 | 380 | 13, 5 | 88 | 2 | 9 | 2, 0 |
| 54 | 18 | 370 | 12, 8 | 89 | 4 | 7 | 1, 5 |
| 55 | 14 | 352 | 12, 6 | 90 | 1 | 3 | 0, 5 |
| 56 | 13 | 338 | 12, 4 | 91 | 2 | 2 | 0, 0 |
| 57 | 8 | 325 | 11, 9 | 92 | — | 0 | —— |

From this table we gather the sad certainty of the short career of those who practice the medical art. Supposing the ordinary duration of human life to be 70 years, we see that scarcely a fourth part of our medical brethren attain that age; and hardly 1 in 15 reaches the age of 80. Or again: if in youth the resolution be taken to devote oneself actively to the laborious pursuits of medical science and practice, in order to enjoy in old age the fruits of a life of exertion, it is a melancholy fact, that one-half the number of practitioners is cut off before that period: so that the remarks of Jean Paul are peculiarly applicable to our case. " The life of man," says this author, " has often been compared to that of travellers or pilgrims: it seems to me rather to resemble that of an innkeeper, who is ever busy about his guests, receiving them, and seeing them depart; and who, on the occurrence of every interval of unprofitable repose, longs for a fresh bustle, desirous of more work when at rest, and of rest when at work; always hoping for the time to come when quiet and ease will suffer him to enjoy his arm-chair in tranquility; but, in general, before that time arrives, he is gathered to his eternal rest."

There are a few points of comparison between the mortality in the medical and that in other professions, which I cannot willingly omit to notice. Divines, (*les theologiens*,) among all the professions, are those who seem to stand the best chance of long life; and if *they* die off more slowly, or enjoy more longevity than the other, it is just the reverse with medical practitioners. The following summary will show the difference:—

| AGES. | DEATHS. | |
|---|---|---|
| | Physicians. | Divines. |
| 23 to 32 | 82 | 43 |
| 33 42 | 149 | 58 |
| 43 52 | 160 | 64 |
| 53 62 | 210 | 182 |
| 63 72 | 228 | 328 |
| 73 82 | 141 | 255 |
| 83 92 | 30 | 70 |
| | 1000 | 1000 |

And the following list may serve to show how much shorter the probable length of life is among medical men than among others. Taking 100 individuals in each calling, the number who attained the age of 70 have been, among

| | |
|---|---|
| Divines, | 42 |
| Agriculturists and foresters, | 40 |
| *Employes* in high offices, | 35 |
| Mercantile persons and traders, | 35 |
| Military men, | 32 |
| *Employes* in lower departments, | 32 |
| Advocates, | 29 |
| Artists, | 28 |
| Teachers, Professors, | 27 |
| Medical practitioners, | 24 |

But what are the causes to which we must attribute this low place in the scale of vitality held by our profession? It would be needless to enumerate them to those who are familiar with the extent of labor which the practice of medicine entails. There is, perhaps, no profession which requires more moral and physical exertion than ours, which allows less of repose, or of that tranquility which is so conducive both to internal and external life. There is none which exposes the members of it to such bodily fatigue, such mischief from bad weather—such disturbance of the night's rest,—so much watching, irregularity of meals, disorders of the digestive organs, and mental affections of all kinds: in a word, to such dangerous influences, perpetually recurring, and all tending to sap the vital powers. If we add to this; that there are far more practitioners cut off by contagion than are commonly supposed, we shall easily see the groundlessness of the satirical observation, that *sybaritism* is the rock on which medical men are wrecked. It may be mentioned, that in the table given above, the great majority of those who were the subjects of it were country practitioners, whom to accuse of luxurious living were too bitter a satire,—one, indeed, wholly undeserved by a profession, which has such strong claims to public gratitude, since those who practice it abridge at least, if they do not absolutely sacrifice their existence, in endeavoring to prolong that of others.—*Medical Gaz., from Berliner Medicinische Zeitung, and Annales d'Hygiene.—Ibid.*

17. *Austrian Statistics.*—In the year 1833, the number of deaths in the Austrian monarchy was 665,731, being 76,917 fewer than in the preceding year; the deaths from cholera, however, in the latter year, may account for the difference. The number of births was 815,293. Among the deaths were by suicide, 724; hydrophobia, 35; casualities, 503; murdered, 422, (in the preceding year, 466;) executed, 36, (in the preceding year, 53.) There were 450 persons who were above 100 years of age. The population of Austria, including Lombardy, Venice, Dalmatia, the Tyrol, etc. is at present reckoned at about 34,000,000. —*London Medical Gazette, Jan.* 1835.—*American Jour. Med. Sci. May*, 1835.

18. *Method of Obtaining the Separation of a Necrosed Sequestrum without an Operation.*—It is stated by Dr. Bouget, that as early as 1814, Professor Delpech, having been disappointed in the treatment of some cases of necrosis, resorted to the plan of dissolving the calcarious matter of the bone by the application of sulphuric acid, in order to soften its texture sufficiently to render it susceptible of being cut with a knife or cutting forceps. A young man whose arm had been amputated, experienced two very severe attacks of hospital gangrene, which occasioned the end of the bone to project an inch and a half beyond the stump. Months probably would have been consumed in the separation of this sequestrum; but this delay Professor Delpech effectually obviated. He had the external surface of the denuded bone covered with a pledget of lint dipped in a solution of diluted sulphuric acid. A ball of charpie, wet with the same fluid, was likewise introduced into the medullary canal, the reticulated texture of which had been previously broken down. In twenty-four hours the portion of bone was found sufficiently softened to admit of its being separated with ease, and in ten days the end of it was covered with healthy granulations, after which a cure was speedily accomplished.

In 1816, an individual presented himself at the Clinique at Montpellier, affected with a necrosis involving nearly the whole extent of the tibia. Although he was possessed of a good constitution, and was in a condition to undergo a formidable operation, M. Delpech resolved to adopt the procedure by which he had succeeded in the above case. He was desirous at the same time of accomplishing this object without the necessity of dividing the integuments by instruments. To effect this, an eschar was made with caustic potassa upon the upper part of the leg of the extent of six francs, and after this eschar was detached from the bone, a pledget of lint dipped in diluted sulphuric acid was applied to it, which, after two or three dressings, renewed at intervals of six hours, became so soft, that it could be broken down with common dressing forceps. Two succeeding applications of caustic potassa and sulphuric acid were then made lower down, in a line with the first, and in this manner the sequestrum was exposed to the extent of five or six inches in length by one and a half in breadth. It was extracted with great facility, measured more than six inches in length, and represented nearly two-thirds of a cylinder. The patient experienced but very little pain, and was discharged cured in a month after his admission.

Dr Bouget represents that M. Delpech had pursued the same practice with equal success in several other cases, both in hospital and civil practice, and that he had himself resorted to it with great advantage in a case of necrosis of the tibia in a child.—*Jour. des Connais. Medicales, Jan.* 1835.—*N. A. Archives, Aug.* 1835.

19. *Nux Vomica in Spasmodic Asthma.*—A case is reported in Kausch's *Geist und kritik. der medec. chirurg. Zeitschriften*, of a young man, aged 20 years, who, after having employed all the most efficacious remedies for spasmodic asthma without success, was effectually relieved by the continued use of nux vomica. The habitual difficulty of respiration subsided, and the paroxysms of asthma disappeared and did not return.—*Strohmayer Medecinish. Prak. Darstellung*, 1831.—*Ibid.*

20. *New Method of Obliterating Aneurismal Tumors of the Extremities.* By M. Leroy D'Etoille.—The procedure proposed consists in establishing mediate compression upon two points of the artery, at an interval of two inches from each other. The portion of blood thus isolated, as it were, coagulates more promptly, than when compression is made in the ordinary way upon a single point. In order to promote the formation of a coagulum, M. Leroy keeps the part covered with ice, and with the view of coagulating the albumen of the isolated portion of blood, he employs acupuncture and galvanism.—*Lancette Francaise, Mars*, 1835.—*Ibid.*

21. *Lepra Vulgaris.*—The editor of the London Medieal and Surgical Journal remarks:—we have lately cured a young woman of lepra vulgaris, of five years standing, which covered the whole cutaneous surface, and defied a variety of remedies, with the following ointment. Her general health was duly attended to.

℞ Hydriodate of potass, ʒ iss.

Prepared lard, ℥ iss.

Tincture of opium, ʒ i.

M. Gibert found the following successful at the Hospital St. Louis:

℞ Ioduret of Ammonia, ℈ i.—ʒ i.

Prepared lard, ℥ i.

Daily for twenty-nine days.—*Ibid.*

22. *Remedy for Itch.*—Dr. Hospital uses the following ointment with success:

℞ Lac. Sulph. ℥ iss.

Pulv. Clor. Calcis. ℥ ii.

Adipis, ℥ vi.

Double this quantity effects a cure.—*London Med. and Surg. Jour. May*, 1835.—*Ibid.*

23. *Cure of Jaundice.*—Frere Le Ome used to cure jaundice by giving a drachm of dried and powdered walnut leaves, infused in a glass of white wine every morning, fasting. From fifteen to twenty doses were for the most part sufficient to annihilate the disorder.—*Ibid.*—*Ibid.*

24 *Newly discovered arrangement of the Arteries in the Erectile Tissue of the Penis.* By Professor J. MULLER, of Berlin. (*Archiv fur Anatomie und Physiologie*, Number II. for 1835.) The second number of Muller's (late Meckel's) Archiv contains the account of an interesting discovery recently made by the editor, Professor J. Muller, of Berlin, relating to the disposition of the small arteries of the penis, which tends to explain the structure of the erectile tissue of that organ, a subject which, notwithstanding the investigations of Cuvier, Tiedemann, Moreschi, and Panizza, was still involved in much obscurity. These observations point out a fact entirely new in the structure of the arteries of the erectile texture, and promise to throw some light on the nature of the erectile condition of the blood vessels.

Most of those who have investigated the structure of the erectile textures of the penis by injections, have contented themselves with filling the veins of the organ, and thus, although the structure of the venous caverns of the *corpora cavernosa*, and the dilated veins of the *corpus spongiosum urethræ* was sufficiently well understood, yet very little was known respecting the mode of termination of the smaller arteries. It has been generally believed that the same small capillary arteries which nourish the penis carry the blood into capillary veins, that the blood passes from these into the dilated venous branches or sinuses, and that the state of erection depends on the retardation of the flow of blood in these venous spaces.

Professor Muller has, by a careful injection of the arteries of the penis, pointed out, besides the capillary branches which nourish the penis and transmit the blood into the capillary vessels and dilated veins, a number of very remarkable appendices connected with smaller arteries, both in the *corpora cavernosa penis* and *corpus spongiosum urethræ*, which, from several circumstances, it is very probable, are the vessels more immediately concerned in maintaining an increased quantity of blood in the penis during erection.

The easiest way of rendering these two sets of arterial branches apparent, is to inject the principal artery of the penis, before its subdivision, with size and vermillion of moderate consistence, and then, making a longitudinal section of one of the *corpora cavernosa*, to wash away any part of the injected mass which may have passed into the venous spaces. The ramification of the nutrient arteries will then be easily seen upon the inner sides of the venous spaces, the arteries becoming smaller and smaller, until at last they pass into the minute capillary network, the branches of which cannot be seen with the naked eye. Besides these nutrient ramified arteries, there will also be seen upon a careful examination another set of arterial branches of a different size, form and disposition, which are given off nearly at right angles from both the larger and smaller trunks of arteries. These arterial processes are about one-hundredth of an inch in diameter, and one-twelfth long, and are quite easily seen with the naked eye. They project into the cavities of the spongy substance, and terminate either bluntly or by dilated extremities, without undergoing any ramification. These short arterial processes are turned round at their extremities into a semi-circle or more, and present a contorted appearance, which circumstances has suggested to Professor Muller the name of Helicine arteries, which he has applied to them.

The helicine arteries of the penis are more easily seen in man than in any other animal which Professor Muller has examined. He has found them in all the animals in which he has sought for them; they are to be seen at the posterior part only of the penis in the stallion, but in the dog exist throughout the whole organ.

In man, the helicine twigs of the penal arteries sometimes come off singly, and at other times they form tufts or bunches, consisting of from three to ten branches, and having in general a very short common stem. The swelling at the extremity, when it occurs, is gradual, and is greatest a little way from the end. The helicine branches given off from large arteries are not of a greater size than those coming from smaller ones, and even the smallest capillary arteries of the *profunda penis*, which can be seen with the help of a glass only, give off helicine twigs of a much greater size than themselves.

Each helicine branch projecting into a venous cavern is covered by a thin membrane which Professor Muller regards as the inner coat of the dilated vein, and when there is a tuft of helicine twigs, the whole tuft is covered with one envelope of a gauze-like membrane. This covering is considerably thicker on the helicine arteries in the posterior part of the *corpus spongiosum urethræ* than in the *corpus cavernosum*, but it seems probable that this is in some measure connected with the state of reple-tion of the arteries, for when the injection has run very well, it becomes difficult to distinguish the external covering.

Professor Muller states, that he could not discover any apertures either in the sides or in the ends of the helicine arteries, but he seems to regard it as probable that minute apertures do exist, which may be of a nature to allow the passage of blood in some states and not in others.

The helicine arteries are not, as some might be inclined to suppose, loops of vessel which have been incompletely filled, and which, after making a coil, pass into venous spaces, as E. H. Weber discovered to be the case with the arteries of the maternal portion of the placenta; they are merely projecting branches from the arterial trunks containing blood.

The helicine arteries are more numerous towards the root than near the point of the penis. They exist in the *corpus spongiosum urethræ* especially towards its bulb, but they are not so easily seen there as in the *corpora cavernosa*. They have not yet been observed in the glans. Their structure is nearly the same in all the animals in which they have been observed; those of the ape bear the nearest resemblance to those of man, and in most animals they are less obvious than in the human subject. In the horse and dog, they give off small nutrient twigs from their sides, which render them more difficult to be seen in these animals than in man.

Professor Muller conceives these helicine of tendril-like arteries to have an intimate connection with the process of erection, and there is every probability that this is the case; but experiments and observations are still wanting to show in what manner these arterial branches are affected in the erected and non-erected condition of the texture in which they exist.

We recommend to the attention of our readers this discovery of Professor Muller's, whose researches have already done a great deal for the advancement of physiological anatomy. The memoir is accompanied with a well executed engraving, containing numerous representations of the helicine arteries, which we regret that time has not enabled us to copy.—*Ed. Med. and Surg. Jour. July*, '35.

25. *Abnormal Secretion of Milk from the Scrotum.*—The following very extraordinary case is contained in the inaugural thesis of M. F. Koller, the first thesis presented to the University of Zurich. There is perhaps nothing similar to it to be found in the annals of the science, if we expect a case somewhat analagous, cited in *Hufeland's Journal*, Vol. 54, No. 2. The milky nature of the fluid secreted, was put out of all doubt by microscopic observations and chemical analysis. It was found to contain all the characters of milk. and its production was evidently connected in some way or another with the exanthema and ulcer of the right lower extremity. The nature of the stools and urine was natural throughout the whole period during which the patient was under observation. The case is taken from the *Gazette Medical de Paris* of Saturday last, July 4:—

H. V., 21 years of age, had enjoyed good health up to the age of 15, when he began to complain of some feverish symptoms. A few days afterwards the left lower extremity presented an eruption of small, slightly elevated red spots, occasioning burning heat and pain in the skin: these symptoms soon disappeared, but the foot began to swell, and the lower extremity became tumefied and soon acquired double its natural volume. The general health of the patient was now in no way influenced by the disease. The tumefaction emptied itself from time to time by two small orifices below Poupart's ligament, giving exit to a white transparent vicid fluid, in greater or less quantity. As the tumour diminished the exanthema reappeared. The patient passed three years in this state, which was not altered by the various remedies employed. At this period the genital organs always little developed and extremely small, began to develope themselves in a sudden and extraordinary manner; the scrotum especially became very large; the external surface of this part was soon covered with a number of small vesicles, which opened, and discharged a great quantity of milky fluid: when the scrotum was compressed, a quantity of the same fluid issued through the two small pores we have noticed, below Poupart's ligament on the inner sides of the thigh. This secretion, though generally of a nature completely milky, sometimes presented an aspect slightly serous. The tumor continued to enlarge, although the secretion was not constant, periodically, every three or four weeks. There were now pains in the groin and lions, darting to the scrotum, and when the patient assumed the upright posture, about two or three pounds of the milky fluid came away. Since the commencement of the disease in the scrotum, the ulcer of the leg had closed, and the tumefaction of the extremity much diminished. On the 23d of August, 1833, the patient was admitted into the hospital at Zurich. The scrotum was now tumefied, and to the touch gave the sensation of a female breast; the skin of the penis was tumefied; the color of the parts was more deep than natural; the corpora cavernosa were small, and retracted towards the pubis; the testicles were also very small, and drawn up to the rings; the larynx was little prominent, but the voice was not feminine; the beard and the hairs on the pubis were delicate and few; the right extremity was a little larger than the left.

During the patient's stay at the hospital, the secretion of milk took place several times. Thus, about the 26th of August, the usual precursory symptoms appeared, and more than three pounds of milk were discharged. On the 30th, there was a new access of fever, and an appearance of the exanthema on the right thigh. On the 11th of September, a fresh discharge of about half a pint of fluid took place, not presenting the same white color or consistence of the former, and giving out a little the odor of semen. During the months of September and October, the tumor of the scrotum and the secretion of a milky fluid alternated frequently with the exanthema on the thigh; however, the tumefaction of the skin covering the genital organs, diminished remarkably, as well as the quantity of fluid discharged, which became more serous and troubled in its appearance, and finally disappeared.

26. *Remarks on the Use of Oiled Silk and Warm Water Dressing.* By Mr. LISTON.—The plaister used by Mr. Liston to recent wounds, such as after amputations, etc., is composed of a spiritous solution of isinglass, spread over oiled silk; the solution is spread, when hot, on strips of the silk, and applied immediately. After amputations, the strips are used of considerable length, so as to give support to the flaps, and an interval is left between each, to allow the escape of matter, should adhesion not take place. Mr. Liston has used it for some years, and has found it to succeed in almost every case. It is easily applied, does not heat the limb, as the usual mode of dressing does, is easily removed in case of secondary hemorrhage, allows the escape of offensive matter, and prevents the fœtor attending the confinement of the discharge; often the wound does not require a second dressing, and the plaister frequently supersedes the necessity of a bandage. The use of warm water dressings, as employed by the same surgeon, consists simply in pledgets of lint, the size of the ulcer, dipped into warm water, and applied to the part, a piece of oiled silk a little larger being placed over it to prevent evaporation. It is found, in some cases, quite effective, without the assistance of any other remedy; occasionally some slight astringent, such as a weak solution of sulphate of zinc or copper is indicated.

Both dressings are cleaner and less painful than any other; the last does not require a sponge; indeed, there have been no sponges used in the hospital for some time, except during operations. Mr. Liston said he had dispensed with them at Edinburgh, as he had seen the indiscriminate use of the same sponge by the various pat ents, attended with the worst consequences. It was often the cause of the spreading of one description of ulcers, of a malignant character, through the wards of the hospital.

It is not for the medicinal use of the water, as stated by a late writer on the subject, that this dressing is applied, it is in place of a poultice. It is by no means a novelty: many surgeons in the army and navy have employed it for years with success.—*London Med. and Surg. Journal, June*, 1835.—*N. A. Archives, Sept.*

27. *Treatment of Recto-Vaginal Communication.* By SIR B. C. BRODIE.—The communication between the vagina and rectum used to be one of the approbriums of our art, the patient's life being rendered miserable, with little or no hopes of recovery. Of late, however, a simple and scientific method of relieving the patient in these cases has been contrived by Mr. Copeland, who has succeeded in curing several patients laboring under it, simply by dividing the sphincter muscle of the anus. The sphincter muscle being divided, the fæces are not retained in the rectum; they run out as fast as they enter it, so that the bowel is kept empty and contracted, and altogether in a passive state, and the communication between the rectum and vagina is thus enabled to cicatrize. I do not know whether this would answer if the communication were of large size, but I am told that it has answered very well in the cases in which Mr. Copeland has hitherto employed it. I cannot but regard the application of this operation to these cases as one of the principal improvements of modern surgery; and the simplicity of the practice forms one of its principal recommendations. Of course it can be recommended in those cases only in which, independently of the opening into the vagina, the parts are in a healthy state.—*London Med. Gaz., June*, 1835.—*Ibid.*

28. *Camphorated Citrine Ointment in Hydrocele.*—A few weeks after a fall which occasioned an inguinal hernia, the right testicle, without any evident cause, began to swell, and became too painful to be handled. (The accident occurred to our correspondent.) This was soon followed by hydrocele, when the pain, which had by this time abated considerably, went off gradually, but the swelling seemed to be stationary. Meanwhile the hydrocele increased, and caused much suffering from tension of the scrotum. I consented to have the water drawn off, but in the interval a different mode of treatment occurred to my mind. Having frequently witnessed the beneficial effects of the citrine ointment, (*Unguentum Hydrargyri Nitratis*,) with camphor in cases of glandular and other indurated tumors, I thought it would also excite the torpid absorbents in those of an aqueous kind, and therefore preferred this trial to that of the knife. In applying it, I grasped the left or sound side of the scrotum firmly, so as to make the tumor as tense as possible, and then rubbed a small quantity of the ointment (about 25 or 30 grains) over its surface for a due length of time. This I repeated every evening as long as it was necessary, generally before a fire. After using the ointment for a week or ten days, the tumor was evidently diminished in bulk, and, persevering with it some time longer, the tumor eventually disappeared. In the meantime there was little alteration in the swelling of the testicle, but as it gave me little or no pain, except when handled, I left it to its own vis medicatrix, and that was slow enough, as it was fully three months before it was reduced to the size of the other. The hydrocele has never returned in any way. The citrine ointment was made by adding a drachm of camphor to an ounce of the ointment.—*London Lancet, May*, 1835.—*Ibid.*

29. *Catechu in Mercurial Pytalism.*—Capt. B., twenty-five years of age, having had occasion to undergo a short course of mercury, whilst his regiment was encamped near Harwich, during rather a cold summer, unexpectedly experienced, about the fifth day of the course, approaching pytalism; and when I was summoned to visit him in the evening, I found him in bed, spitting, or rather slavering, profusely, and presenting a picture of despair, from distress of mind, occasioned by his situation at a moment when he was daily expecting a visit from a party of valued friends, most of them females, to the camp. It was no easy matter to determine, under these urgent circumstances, what means to use. I had long ago proved the utter inefficiency of alum, opium, and other reputed antidotes against salivation, and at last I thought of catechu, of which I had made a strong decoction for some other purpose, though it was a remedy as yet untried, at least by me.

To be brief, I desired my patient to take a wine-glass full (about ℥ii. to ℥iiss.) of this decoction every two hours, or even oftener, if his stomach would bear it; and also to gargle his mouth and fauces as frequently as possible with the same. The result was, that when I visited him early the next morning, the spitting had entirely ceased, and every other sign of the effects of mercury had wholly disappeared, debility from the pytalism alone excepted. I think that since this case occurred I have had further proof of the efficacy of catechu in obviating the ultimate effects of mercury, and I invite your numerous readers to make trial of it in cases of mercurial pytalism. Instead of ʒiiss. of catechu to half a pint of boiling water, according to the London Pharmacopœia, I use at least ʒiij. to that quantity of boiling water; for whether infusion or decoction be used, it ought to be made as strong as possible.—*Ibid.*—*Ibid.*

